# GPR182 is a broadly scavenging atypical chemokine receptor influencing T-independent immunity

**DOI:** 10.3389/fimmu.2023.1242531

**Published:** 2023-07-24

**Authors:** Serena Melgrati, Oliver J. Gerken, Marc Artinger, Egle Radice, Martyna Szpakowska, Andy Chevigné, Giulia D’Uonnolo, Paola Antonello, Sylvia Thelen, Pawel Pelczar, Daniel F. Legler, Marcus Thelen

**Affiliations:** ^1^ Institute for Research in Biomedicine, Università della Svizzera italiana, Bellinzona, Switzerland; ^2^ Graduate School for Cellular and Biomedical Sciences, University of Bern, Bern, Switzerland; ^3^ Biotechnology Institute Thurgau (BITg), University of Konstanz, Kreuzlingen, Switzerland; ^4^ Immuno-Pharmacology and Interactomics, Department of Infection and Immunity, Luxembourg Institute of Health (LIH), Esch-sur-Alzette, Luxembourg; ^5^ University of Basel, Center for Transgenic Models, Basel, Switzerland; ^6^ Faculty of Biology, University of Konstanz, Konstanz, Germany; ^7^ Theodor Kocher Institute, University of Bern, Bern, Switzerland

**Keywords:** GPR182, chemokine, atypical chemokine receptor, signal transduction, marginal zone

## Abstract

Immune responses highly depend on the effective trafficking of immune cells into and within secondary lymphoid organs (SLOs). Atypical chemokine receptors (ACKRs) scavenge chemokines to eliminate them from the extracellular space, thereby generating gradients that guide leukocytes. In contrast to canonical chemokine receptors, ACKRs do not induce classical intracellular signaling that results in cell migration. Recently, the closest relative of ACKR3, GPR182, has been partially deorphanized as a potential novel ACKR. We confirm and extend previous studies by identifying further ligands that classify GPR182 as a broadly scavenging chemokine receptor. We validate the “atypical” nature of the receptor, wherein canonical G-protein-dependent intracellular signaling is not activated following ligand stimulation. However, β-arrestins are required for ligand-independent internalization and chemokine scavenging whereas the C-terminus is in part dispensable. In the absence of GPR182 *in vivo*, we observed elevated chemokine levels in the serum but also in SLO interstitium. We also reveal that CXCL13 and CCL28, which do not bind any other ACKR, are bound and efficiently scavenged by GPR182. Moreover, we found a cooperative relationship between GPR182 and ACKR3 in regulating serum CXCL12 levels, and between GPR182 and ACKR4 in controlling CCL20 levels. Furthermore, we unveil a new phenotype in GPR182-KO mice, in which we observed a reduced marginal zone (MZ), both in size and in cellularity, and thus in the T-independent antibody response. Taken together, we and others have unveiled a novel, broadly scavenging chemokine receptor, which we propose should be named ACKR5.

## Introduction

Leukocyte trafficking during homeostasis and inflammation is orchestrated by chemokines binding to their cognate receptors (1). Chemokines are small (8-10 kDa) chemotactic cytokines (2) that can bind multiple canonical receptors and can drive cell migration, or atypical chemokine receptors (ACKRs), which mainly act as scavengers. ACKRs fail to induce G-protein mediated signaling upon ligand binding (3), but rather take up and remove chemokines from the extracellular milieu, thereby generating fine-tuned chemokine gradients necessary for migration of multiple cell types.

The ACKR family currently comprises four members: ACKR1 – ACKR4, each with unique binding abilities (2). All known ACKRs, except for ACKR1, fall into the γ-subfamily of rhodopsin like G-protein coupled receptors (GPRCs) together with canonical chemotactic receptors (4). ACKR1 expressed on erythrocytes scavenges a variety of chemokines, but also transcytoses chemokines across endothelial cells (3, 5, 6). ACKR2 internalizes and degrades multiple inflammatory chemokines of the CC family and plays a critical role in the dissolution of inflammation (7–9). ACKR4 binds the T-cell chemoattractants CCL19, CCL20, CCL21, CCL22, CCL25 (10–12). The third member, ACKR3, also known as CXCR7, binds and scavenges CXCL12, and with lower affinity, CXCL11 (13, 14). The closest relative of ACKR3 by phylogeny is GPR182 (4, 15, 16). GPR182 was initially suggested to bind adrenomedullin, however this was later refuted (17, 18). Recently, two groups have shown that GPR182 can bind CXCL9, CXCL10, CXCL12 and CXCL13 (19, 20). However, the full breadth of chemokine binding and scavenging remains to be confirmed.

The details of how ACKRs perform their scavenging function are not fully understood. Typically, upon ligand binding, ACKRs internalize into endosomes. Chemokines are then sorted for lysosomal degradation, while the receptor is recycled back to the plasma membrane (21). This scavenging activity is thought to require the phosphorylation of the receptor C-terminus by G-protein coupled receptor (GPCR) kinases (GRKs), which leads to β-arrestin recruitment (22–25). However, whether recruited β-arrestins are required for scavenging is disputed (26, 27). Moreover, whether internalization and scavenging by GPR182 depends on typical C-terminus phosphorylation by GRKs is unknown.

GPR182 has been shown to be expressed in the endothelium of mammals and zebrafish (19, 28–33). In mice GPR182 is expressed in the endothelium of multiple organs, such as spleen, liver, lymph nodes; including tumor-associated lymphatics, and also intestinal stem cells (20, 31, 34). Our group has previously shown that GPR182 is also very broadly expressed in splenic vasculature (30), although the role for GPR182 in this setting is currently unknown.

ACKR3 and ACKR4 were shown to be expressed in the splenic vasculature (35, 36). In particular, ACKR4 is expressed in the peri-marginal sinus, where it controls marginal zone architecture and T cell homing into follicles. ACKR3 is largely found in red pulp sinusoids including the peri-marginal sinus but also on marginal zone B cells (MZB). B cell-specific expression of ACKR3 is necessary for marginal zone formation and function, namely response to the T-independent antigen TNP-Ficoll (37). In addition, systemic inhibition of ACKR3 caused marginal zone disruption (38), i.e. the reduction in MZB numbers, as well as systemically increased levels of CXCL12.

The marginal zone (MZ) is a specialized area of the spleen which surrounds B cell follicles, located on the border between the white pulp and red pulp MZ B cells (MZBs) that can capture blood-borne antigens to initiate an immune response (39). MZB cells are characterized by the expression of IgM^Hi^ IgD^Low^ CD21^Hi^ CD1d^Hi^ CD23^-^ and are generated from transition stage 2 B cells in spleen follicles (40). MZBs possess the capacity to respond to T cell-independent type II antigens (TI-II) such as trinitrophenol Ficoll (TNP-Ficoll), a hapten-conjugated particulate polysaccharide. In the presence of C3d complement, MZBs bind these antigens via their complement receptors CR2 and CR1 (CD21/35) (41). MZB then shuttle to follicular dendritic cells (FDCs) inside the B cell follicle to deliver the antigen (42). To attract MZBs, FDCs secrete CXCL13, which guides CXCR5-mediated migration. MZBs can return to the MZ following sphingosine-1-phosphate (S1P), of which high levels are found in the blood (43). While follicular B cells (FoBs) are dragged into the blood when migrating towards S1P, MZBs are retained via integrin-mediated adhesion to MZ reticular cells (44). Whether the MZ is properly functional in GPR182-KO mice, which display elevated levels of CXCL13 in the serum, is not yet known (19).

Here we extend recent evidence that GPR182 is a chemokine scavenger, with a profoundly broader binding spectrum than previously anticipated. We show that GPR182 is able to spontaneously traffic between the plasma membrane and endosomes, in a β-arrestin-dependent manner. However, β-arrestin recruitment, internalization and scavenging does not entirely rely on the C-terminus of the receptor. We show that CCL28 and CXCL13, which are not scavenged by any other ACKR, can be taken up and removed from the extracellular space by GPR182, both *in vivo* and *in vitro.* We also show that GPR182-KO mice develop smaller splenic marginal zones, both in size and cellularity, leading to altered responses to TI-II antigens.

## Methods

### Cell lines, cloning and transfection

Mouse 300.19 pre-B cells were (preferably used for flow cytometry) cultured in B cell medium containing RPMI-1640 supplemented with 10% FBS, 1% PenStrep, 1% nonessential amino acids, 1% Glutamax, and 50 μM β-mercapto ethanol (β-ME). All cell culture media and supplements were from Gibco/Thermofisher. Cells stably expressing ACKRs or GPR182 were transfected using Amaxa Nucleofector (Lonza) with 3 μg DNA according to the manufacturer’s instruction. Receptors were expressed fused via a self-cleaving peptide to green fluorescent protein (GFP) using the pME-Cas9-T2A-GFP plasmid (#63155 – Addgene). Receptors and GFP are post-translationally split to produce the two proteins marking cells and their receptor expression level by GFP fluorescence intensity (45).

Adherent cells were preferably used for confocal microscopy. HeLa wildtype cells were cultured in DMEM (Pan Biotech, Aidenbach, Switzerland) supplemented with 10 % FCS (Lonza, Basel, Switzerland) and 1 % PenStrep (Pan Biotech). Cells were transiently transfected using the Neon Transfection System (ThermoFisher, Waltham, MA, USA) according to the manufacturer’s protocol. Cells were cultured in DMEM supplemented with 20 % FCS for 44-48 h. For split-luciferase assays, plasmid ratio was 1:1 and for the mini G protein activation assay, the ratio was 3:1.

HEK293 cells were cultured in DMEM supplemented with 10 % FBS and 1 % PenStrep (all from Gibco). HEK293^WT^, β-arrestin 1^KO^, β-arrestin 2^KO^, β-arrestin 1,2^KO^ were obtained from Stéphane Laporte (McGill University), and transiently transfected with a pALPs plasmid containing a construct in which mGPR182 was linked to Venus fluorescent protein through a (SGGG)_3_ sequence using Amaxa Nucleofector (Lonza) with 3 μg Ingenio Electroporation Solution (Mirus Bio, Cat: MIR 50111). The ΔC-constructs were generated by PCR deleting the entire cytosolic C-terminus after the end of the predicted helix VII (NPILYNFLS) and re-ligation into pALPS-Venus. β-arrestin rescue experiments were performed by transfecting 2 μg plasmid containing β-arrestin 1 linked to ECFP and/or 0.5 μg plasmid containing β-arrestin 2 linked to mCardinal. G protein-coupled receptor kinase (GRK) knockout cells were previously generated (46) and kindly provided by Asuka Inoue (Tohoku University). The generation of pcDNA3 LgBIT-miniGα_i_ (47), pIRES Gα-nLuc Gβγ-cpVenus (23), pcDNA3-nLuc11 (48) and pcDNA3 β-arrestin1A-nLuc156 and pcDNA3 β-arrestin2i1-nLuc156 (23), has been previously described. The ST/A variant was synthesized by Twist Biosciences, substituting all codons for serine and threonine to codons for alanine in the second intracellular loop, after the DRY motif, resulting in a change in amino acid sequence from DRYVTLTSASPS to DRYVALAAAAPA (position 150-161 in amino acid sequence).

### Chemokine expression and purification

Recombinant chemokines were expressed, purified, and fluorescently labelled as previously described (12, 49, 50). In case of high sequence homology (hCXCL12) or good cross reactivity (mCCL25) was observed only human or mouse chemokines were expressed and used.

### Binding and uptake assays by flow cytometry

All binding and uptake experiments were performed at 4°C or 37°C for 45 minutes. Mean fluorescence intensity was measured by flow cytometry. When quantifying, MFI from non-transfected parental cells was subtracted.

For binding and uptake assays, 300.19 pre-B cells stably transfected with receptors (and GFP in a T2A system), or parental, were incubated with the indicated concentrations of chemokines (all labeled in AF647 or Dy649) for 45 minutes. Cells were washed and analyzed by FACS (Fortessa, BD). The MFI of GFP, which is proportional to the level of receptor expressed (45), was used for normalization.

Direct binding was measured by FACS incubating 300.19 cells expressing GPR182 T2A GFP with increasing concentrations of fluorescently labeled chemokine. Competition binding and scavenging was measured by FACS incubating 300.19 cells expressing GPR182-GFP with a fixed amount (3 nM) of fluorescently labeled chemokine and increasing concentrations of unlabeled chemokine. After 45 minutes cells were washed and analyzed by FACS (Canto I, BD).

### Immunofluorescence and uptake assays by confocal microscopy

HEK293^WT^; HEK293 β-arrestin 1^KO^; β-arrestin 2^KO^; β-arrestin 1, 2^KO^ cells transfected with receptors (and GFP in a T2A system or with an mGPR182-venus construct), were grown on MatTek slides. These were incubated with 100 nM chemokines (all labeled in AF647, ATTO565 or ATTO700) for 45 minutes at 37°C. Cells were washed with PBS, fixed in 4% paraformaldehyde (PFA), permeabilized, stained with antibodies, and mounted in either Fluoromount (Sigma, Cat: F4680) or DAPI-containing Duolink *in situ* mounting medium (Sigma, Cat: DUO82040). Membranes were visualized either by staining with MemBrite Fix Cell Surface Staining Kit (Biotium) following manufacturer’s instructions, or by transiently transfecting cells with a plasmid expressing the myristoylation/palmitylation motif from the Lck fused to the N-terminus of mCherry generating a plasma membrane marker (13).

For live imaging experiments, HEK293 expressing mGPR182 GFP were grown on MatTek slides. On the day of the experiment, complete medium was replaced with Optimem (Gibco) containing 10% FBS. CCL20-AF647 was added dropwise during live acquisition at t = 1 minute, at a final concentration of 200 nM. Live imaging was performed using a Leica SP5 confocal microscope. Chemokine uptake was quantified using ImageJ by measuring Integrated Density (set to threshold) on selected ROIs.

### Calcium microscopy

300.19 pre-B cells expressing CXCR4, CCR7, mGPR182 or hGPR182 were loaded with 1 μM Fura2-AM, and plated on MatTek glass-bottom dishes in MSB buffer. Intracellular calcium rise was measured ratiometrically at 37°C using a Zeiss microscope equipped with a TIL-photonics dual wavelength Polychrome V excitation system before and during chemokine addition (50 nM), and during CaCl_2_ (3.5 mM) and Ionomycin (2.5 μM) addition. Signal intensity of 15 single cells was quantified using ImageJ.

### Immunoblotting

To determine CXCL13 degradation, HEK293 cells expressing either mGPR182 or hGPR182, or primary LECs (isolated as described in (30, 51) were pre-incubated with 50nM Bafilomycin A1 (Baf A1, ThermoFisher) in DMSO or DMSO alone (control) for 1h. Cells were then incubated with 30-100 nM chemokine for 90min. Following two washes, cells were allowed to degrade chemokine in presence or absence of Baf A1 for 90min. Cells were washed in PBS, then lysed in buffer containing 50 mM Tris-HCL pH 6.8, 150 mM NaCl, 1% Tx-100, 1 mM EDTA, 1X Complete (Roche). Samples were loaded on 12% SDS-PAGE gel, and transferred on MeOH-activated PVDF membranes in CAPS buffer (pH 10.5).

ERK phosphorylation upon stimulation was determined in 300.19 pre-B cells expressing CXCR4, CXCR3, mGPR182 or hGPR182 as previously described, using 50 nM CXCL12 or CCL19 and stimulating for 1 min at 37°C (52). Enhanced chemiluminescence was recorded using a Fusion FX (Vilber). If necessary, membranes were stripped using a 0.2M NaOH solution for 5min, and reprobed with a total ERK antibody as described above. Signal intensity was quantified using ImageJ and normalized to total ERK2 signal.

### BRET and split-luciferase assays

For BRET and split-luciferase assays, transfected HeLa cells were washed, transferred in quadruplicates into a 96-well half-area plate (PerkinElmer, Waltham, MA, USA) and loaded with 5 μM coelenterazine H (Biosynth, Staad, Switzerland) at 37°C in a Spark 10M multiplate reader (Tecan, Männedorf, Switzerland). Luminescence (385–440 nm, 350 ms integration time) and additionally for BRET assays, cpVenus fluorescence (505–590 nm, 350 ms integration time), was measured for 10 min followed by stimulation with 300 nM of the indicated chemokine. Obtained data were normalized to PBS alone.

Chemokine-induced β-arrestin-2 recruitment to GPR182, CXCR4 and two chimeras corresponding to GPR182 (1-320) with the C-terminal tail of CXCR4 (303-352) or CXCR4 (1-302) with the C-terminal tail of GPR182 (321-404) was monitored by NanoLuc complementation assay (NanoBiT) as previously described (53, 54). 5 × 10^6^ HEK293T cells were plated in 10-cm dishes and 24 hours later cotransfected with pNBe vectors encoding WT or chimeric receptors C-terminally tagged with nLuc11 and human β-arrestin-2 N-terminally tagged with LgBiT. Twenty-four hours after transfection, cells were harvested and incubated for 15 min at 37°C with coelenterazine-h (Regis Technologies). β-arrestin-2 recruitment to the receptor induced by CXCL12 (or CXCL11) at concentrations ranging from 10 pM to 300 nM was evaluated for 20 min with a Mithras LB940 luminometer (Berthold Technologies). EC50 values were obtained by nonlinear regression curve fitting using GraphPad software analysis.

### Mice

GPR182^mCherry/+^ (GPR182^+/-^) mice were generated by CRISPR/Cas9 genome engineering as previously described (30). ACKR4^GFP/GFP^ (ACKR4^-/-^) were described elsewhere (55).

All animals were healthy and housed in specific-pathogen free facility and all experiments were in accordance with the Swiss Federal Veterinary Office guidelines and authorized by the Animal Studies Committee of Cantonal Veterinary (License: 33/2020; 74/2022). All mice used were in the C57BL/6 background. Animals of both genders were used with no sex-dependent differences observed.

For inhibition of ACKR3, mice were injected subcutaneously with 30mg/kg CCX771 (Chemocentryx) in Captisol or vehicle only. Mice were euthanized 2h later and terminal blood was collected.

For T-independent responses, mice were immunized by intravenous (i.v.) injection with 50 µg TNP-AECM-Ficoll (2,4,6-Trinitrophenyl AminoEthylCarboxyMethyl, BioSearch Technologies, cat F-1300), and blood obtained 4 days later. For analysis of antigen deposition in the follicle, 30 µg TNP-AECM-Ficoll-Fluorescein, BioSearch Technologies, cat F-1300F) were injected i.v., and animals sacrificed 3h later. Spleens were fixed and processed for confocal microscopy as in 2.9. For T-dependent studies, mice were immunized by intraperitoneal injection with NP-KLH (BioSearch Technologies) precipitated in Imject Alum Adjuvant (Thermo Scientific) in a 1:1 ratio. They were sacrificed 14 days later to analyze serum and spleens by ELISA or flow cytometry.

### Organ imaging

Mice were euthanized in CO_2_, and perfused first with 30ml PBS, then with 10ml PBS containing 2% PFA. Organs were harvested and fixed for 4-16 hours in 4% PFA at 4°C on a shaking platform. Organs were washed in PBS and embedded in 3% low-gelling temperature agarose (Sigma, Cat: A9414). Tissues were sectioned using a Leica Vibratome to produce 50-150µm slices. Sections were blocked in Blocking Buffer (PBS, 1% FBS, 0.1% Tx-100, 0.01% NaN_3_) for 1h at RT, then stained with selected primary or directly conjugated antibodies overnight in Blocking Buffer. Following two 20-minute washes, sections were stained using secondary antibodies (1 hour RT) or DAPI (15 minutes RT), washed twice and mounted on glass slides in FluoroMount (Sigma, Cat: F4680). Samples or sections were imaged using a Leica SP5 confocal microscope.

### *In vivo* uptake

Mice were injected i.v. through the tail vein with a 150µl solution containing 2.5 µM fluorescent chemokine(s) in PBS. Animals were euthanized in CO_2_ 30 minutes later, perfused and organs harvested and processed as above for imaging.

### Chemokine ELISA and Luminex

Murine CXCL12 (RnD, cat DY350), CCL28 (RnD, cat DY533) and murine and human CXCL13 were measured by ELISA (RnD, cat DY470, DY801), following manufacturer’s instructions. CXCL13 in cell culture supernatant was measured following overnight incubation of HEK293 cells expressing mGPR182, hGPR182 or WT with 5 nM CXCL13 in complete medium. Murine CCL19, CCL20, CCL21 were measured with a custom Luminex multiplex assay. Sera were obtained by centrifuging clotted blood twice. Spleen and lymph node supernatants were obtained by homogenizing tissues between frosted slides in PBS containing 1X complete protease inhibitors (Sigma, cat 05056489001), and centrifuging twice. Supernatant total protein content was measured by Protein Assay (Bio-Rad) and used to normalize. CCL28 was determined in a suspension of feces (100 mg) diluted in 1 ml PBS.

### Immunization and antibody ELISA

Antigen-specific IgM in the serum was measured by ELISA. ELISA half-area plates were coated with 5 μg/ml TNP-BSA, NP4-BSA or NP20-BSA (diluted in PBS) overnight at 4°C. For total IgM and IgG plates were coated with 2 μg/ml purified rat anti-mouse IgM or IgG. Plates were washed and blocked in 1% BSA-PBS for 2h at RT. Sera were serially diluted in blocking buffer and incubated for 2h at RT. Following washing, alkaline phosphatase-anti-mouse IgM or IgG (SouthernBiotech) was diluted in blocking buffer (1:500) and incubated for 30min at RT. Plates were washed and developed with 1 mg/ml phosphatase substrate (Sigma) in the dark at RT according to manufacturer’s instructions. Absorbance at 405nm was measured every 30 min with BioTek reader.

**Table:** 

Antibody target	Clone	Concentration	Manufacturer	Catalog Number
Anti-B220	RA3-6B2	1:300	BioLegend	103244
Anti-CD19	6D5	1:200	BioLegend	115522
Anti-CD21	7E9	1:200	BioLegend	123406
Anti-CD23	B3B4	1:200	BioLegend	101608
Anti-IgM	RMM-1	1:300	BioLegend	406526
Anti-CD138	281-2	1:200	BioLegend	142512
Anti-CD11b	M1/70	1:200	BioLegend	101212
Anti-CD5	53-7.3	1:200	BioLegend	100632
Anti-Fas	Jo2	1:200	BD Pharmingen	563647
GL7	GL7	1:500	BioLegend	144612
Anti-CXCR5	2G8	1:100	BD Pharmingen	551960
Anti-PD1	RMPI-30	1:200	BioLegend	109117
Anti-ICOS	7E.17G9	1:200	BioLegend	117423
Anti-CD3	17A2	1:200	BioLegend	100204
Anti-CD4	GK1.5	1:100	RnD Systems	FAB554T
Anti-MAdCAM-1	Meca367	1:300	BioLegend	120712
Anti-CXCR4	L276F12	1:100	BioLegend	146505
Anti-IgM AP	Polyclonal	1:500	Southern Biotech	0109-04
Anti-IgG AP	Polyclonal	1:500	Southern Biotech	1030-04
Rat anti-mouse IgM	SB73a	2μg/ml	Southern Biotech	1139-01
Rabbit anti-mouse IgG	Polyclonal	2μg/ml	Southern Biotech	6170-01
pERK1/2	MAPK-YT	1:5000	Merck	M8159
ERK2	1B3B9	1:5000	Merck	05-157
Anti-mCXCL13	143614	1:1000	RnD Systems	MAB470
Anti-hCXCL13	53610	1:1000	RnD Systems	MAB801
Anti-GAPDH	Polyclonal	1:5000	Sigma	G9545
Anti-Rab5	EPR21801	1:100	Abcam	Ab218624
Anti-Lamp1	H4A3-c	1:50	Developmental Studies Hybridoma Bank	

Antibodies for FACS, immunofluorescence, western blot, ELISA.

## Results

### GPR182 is related to ACKRs

Except for ACKR1, which has the least similarity, all known ACKRs (ACKR2-4) fall into the γ-subfamily of rhodopsin like GPRCs together with canonical chemotactic receptors (4). To understand whether GPR182, which is the closest ortholog to ACKR3 in the γ-subfamily, possesses conserved elements with the known ACKRs, we performed alignment analyses. Both human and mouse GPR182 shared elements which were conserved with other ACKRs, for example a slightly modified DRYLAIV motif found in the intracellular extension of the 3^rd^ helix of typical chemokine receptors, which became DRYVTLV in GPR182, and the NPXXY sequence at the end of helix VII (**Figures S1A–C**). The DRYLAIV motif has been proposed to be critical for G-protein coupling but may not be the sole structural component involved (56). We also compared human and mouse GPR182 (**Figure S1D**). The extracellular N-termini which typically comprise the binding site 1 of chemokines (57) showed differences, while binding site 2 composed by the helical transmembrane domains was mostly conserved. The differences in the sequences of the N-termini could indicate a similarity in chemokine recognition but with differences in affinity. Interestingly, at the C-terminus that were divergent, but the serine and threonine residues were mostly conserved among human and mouse GPR182.

### GPR182 interacts with a broad spectrum of chemokines

**Figure 1:**
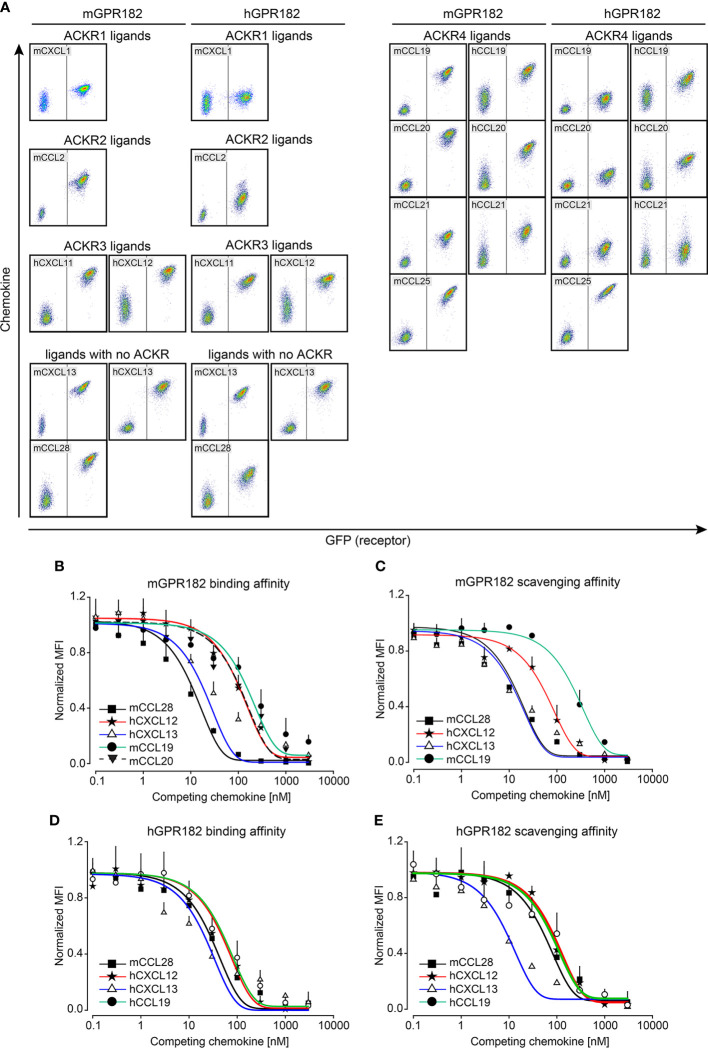
GPR182 binds and internalizes a broad variety of chemokines with low affinity. **(A)** 300.19 pre-B cells expressing mGPR182 T2A GFP (left panels) or hGPR182 T2A GFP (right panels) were incubated (together with parental GFP negative 300.19 cells) with 200 nM fluorescently labelled human or mouse chemokines (as indicated in each panel) at 37°C for 45 min and uptake was measured by FACS. Panels are grouped for shared binding and uptake by other ACKRs as indicated. Shown is one representative experiment out of 5 independent determinations performed. **(B, D)** Competition binding at 4°C or **(C, E)** scavenging at 37°C was measured by incubating 300.19 cells expressing mGPR182 **(B, C)** or hGPR182 **(D, E)** with a fixed concentration of fluorescent chemokine (3 nM) and increasing concentrations of its unlabeled counterpart. (N = at least 3 independent experiments, performed in duplicates. Error bars ± SD).

To determine the ability of murine and human GPR182 (mGPR182 and hGPR182) to take up chemokines, we incubated 300.19 pre-B cells expressing either mGPR182 T2A GFP or hGPR182 T2A GFP, together with parental (GFP-negative) cells with 200 nM or 20 nM fluorescently labelled recombinant chemokines. GPR182-expressing cells were able to take up most chemokines tested, including the previously known hCXCL12 and CXCL13 (both murine and human) (**Figure 1A**, **Figure S2**). In addition, we found that mGPR182 was also able to take up mCCL2 like ACKR2 but only at elevated concentration, hCXCL11 like ACKR3, and the chemokines CCL19, CCL20, CCL21 (both murine and human), mCCL25, known to be scavenged by ACKR4. Moreover, mGPR182 readily scavenged mCCL28, for which no scavenger was described before. hGPR182 was able to internalize the same chemokines except for mCCL19 and mCCL20. Also only minimal uptake of both murine and human versions of CCL21 was observed at elevated concentrations like mCCL2. mCXCL1 was poorly taken up by either murine or human GPR182. To compare binding and uptake capability of mGPR182 with known human ACKR-chemokine pairings, we measured the MFI of 200 nM chemokine bound (**Figure S3A**) or internalized (**Figure S3B**) by mGPR182 and of hACKR3 (in case of CXCL12 and CXCL11) and hACKR4 (in case of murine and human CCL20, mCCL25, hCCL19). Binding and uptake performed by mGPR182 was less efficient in the case of CXCL12, but similar in when CXCL11 was tested compared to ACKR3 and general less prominent than ACKR4 scavenging, with the exception of murine and human CCL20, which are known to be a low affinity pairing for ACKR4 (12). Similarly, we compared human and murine GPR182 efficiencies in terms of binding and uptake (**Figures S3C, D**). Human GPR182 was overall less potent in binding and taking up chemokines, except for mCCL25. It should be noted that uptakes of chemokines measured by flow cytometry could represent an underestimate due to chemokine degradation and dye expulsion by the cells.

### GPR182 is a low-affinity receptor

To further dissect the ability of GPR182 to bind chemokines, we performed a direct-binding experiment using fluorescently labeled mCCL28. We found that mGPR182 preferentially bound mCCL28, with a Kd ~8.5 nM (**Figure S3E**). We then performed competition binding and scavenging experiments, in which we incubated 300.19 cells expressing mGPR182 or hGPR182 with a constant amount of fluorescently labelled chemokine (3 nM) with increasing concentrations of its unlabeled version. We found the highest affinity of mGPR182 for mCCL28 and hCXCL13 (**Figure 1B**, **Table 1A**), compared to other tested chemokines. Similarly, mCCL28 and hCXCL13 displayed the highest scavenging affinity by mGPR182, followed by hCXCL12 and to a lower extent mCCL19 (**Figure 1C**, **Table 1A**). We also confirmed that hGPR182 binds hCXCL13, mCCL28, followed by hCXCL12 and hCCL19, yet overall with lower affinity compared to mGPR182 (**Figure 1D**, **Table 1B**). hGPR182 preferentially scavenged hCXCL13, followed by mCCL28, hCCL19 and hCXCL12 (**Figure 1E**, **Table 1B**). Taken together, these results suggest that GPR182 may be considered a broadly scavenging chemokine receptor which binds and internalizes CXCL13 and CCL28 with the highest affinity, and multiple other chemokines with low affinity.

**Table 1:** Binding and scavenging affinities.

(A) mGPR182 Kd (nM)		
Chemokine	Competition binding	Competition scavenging
mCCL28	10	14
hCXCL13	20	13
hCXCL12	120	60
mCCL20	120	
mCCL19	180	250
(B) hGPR182 Kd (nm)		
Chemokine	Competition binding	Competition scavenging
mCCL28	28	52
hCXCL13	20	9
hCXCL12	50	85
hCCL19	55	75

**Figure 2:**
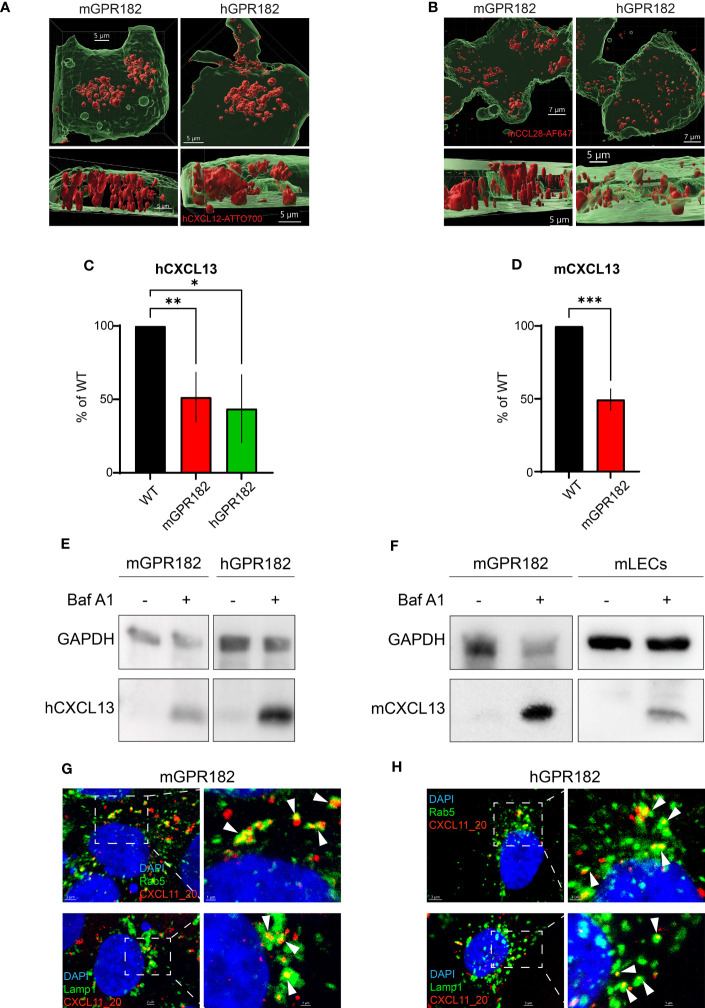
GPR182 is a chemokine scavenger. **(A)** Surface rendering of internalized 200 nM CXCL12-AT700 or **(A)** 200 nM CCL28-AF647 **(B)** (red) in HEK293 cells stably transfected with mGPR182 T2A GFP (left panels, green) or hGPR182 T2A GFP (right panels, green). Scale bars = 5 to 7 μm. **(C)** Remaining hCXCL13 or **(D)** remaining mCXCL13 levels in the cell culture medium following overnight incubation with either HEK293 WT or HEK293 expressing mGPR182 or hGPR182. ELISA measurements were normalized to CXCL13 levels in supernatants of HEK293 WT cells. (N = at least 2 independent experiments, performed in duplicates. Error bars ± SD. Unpaired two-tailed t-test *p<0.05 **p<0.01 ***p<0.001). **(E, F)** Degradation of hCXCL13 **(E)** by HEK293 cells expressing mGPR182 or hGPR182. Degradation of mCXCL13 **(F)** by HEK293 cells expressing mGPR182 or primary mouse LECs, in absence or presence of 50 nM Bafilomycin A1 (Baf A1). Shown are western blots of corresponding cell lysates. GAPDH was used as loading control. **(G, H)** Confocal images illustrating co-localization of CXCL11_20 (red) and Rab5 (top panels) or Lamp1 (bottom panels) in HEK293 cells expressing mGPR182 **(G)** or hGPR182 **(H)**. Panels on the right are magnifications of each image. White arrows point to co-localization of GPR182 and the markers. Scale bars = 3μm [**(G)** top left panel, **(H)** left panels], 2 [**(G)** top left panel, **(H)** left panels], 2μm [**(G)** bottom left panel], 1μm (right panels).

Summary of (A) mGPR182 and (B) hGPR182 binding and scavenging affinities (Kd, expressed in nM) as measured by displacement binding or competition scavenging as shown in **Figure 2**. (N= at least 3 independent experiments, performed in duplicates).

### GPR182 is a scavenger receptor addressing chemokines for degradation

To confirm internalization of chemokines by GPR182, an essential feature of a scavenger receptor, we imaged HEK293 cells expressing murine or human GPR182 expressed in a GFP T2A system following fluorescent chemokine uptake. By 3D reconstruction we could visualize endosomal-like structures containing chemokines, hCXCL12 or mCCL28 (red), within the cell body (green), demonstrating internalization (**Figures 2A, B**). This was also observed in live imaging experiments using HEK293 cells expressing mGPR182 in which endosomes containing fluorescent mCCL20 appear already 4 minutes 30 seconds after chemokine addition (**Video S1**, **Figure S4**). Moreover, GPR182-expressing HEK293 cells were able to markedly reduce hCXCL13 (**Figure 2C**) and mCXCL13 (**Figure 2D**) from the cell culture medium. We then investigated the potential intracellular degradation of CXCL13. ACKR-mediated chemokine uptake into early endosomes leads to acidification of the compartment and separation of the cargo from the receptor. Endosomes containing the receptor can recycle to the plasma membrane, while the endocytosed chemokines are sorted into late endosomes and lysosomes, where the cargo proteins are degraded (58). Indeed, in the presence of Bafilomycin A1, an inhibitor of the proton pump V-ATPase (59), exogenously-added hCXCL13 (**Figure 2E**) or mCXCL13 (**Figure 2F**) were still present in lysates of HEK293 cells (**Figure 2E**) or lymphatic endothelial cells [LECs, which endogenously express mGPR182 (30)] (**Figure 2F**). However, in the absence of the inhibitor, both cell types were able to degrade the chemokine. To further corroborate the fate of GPR182-mediated chemokine internalization, we stained for markers of endosomes and lysosomes (**Figures 2G, H**). We used a GPR182-specific chimeric chemokine (30) to exclude uptake by other typical or atypical chemokine receptors. Indeed, colocalization of the taken up fluorescent chemokine with Rab5, a marker of early endosomes, and the lysosomal marker Lamp1 was visualized, demonstrating that GPR182 is a chemokine scavenger, which is able to deliver chemokines to lysosomes for degradation.

### GPR182 is an atypical chemokine receptor

**Figure 3:**
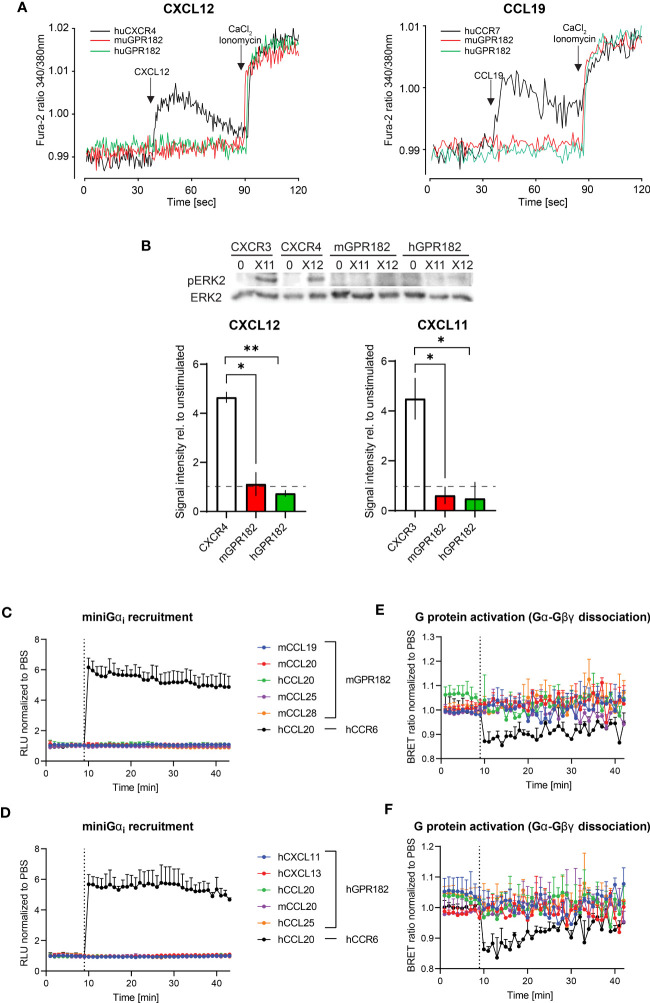
GPR182-chemokine interactions do not induce canonical signaling. **(A)** Stimulation of intracellular free calcium raise by 50 nM CXCL12 (left) and CCL19 (right) in 300.19 pre-B cells expressing cognate canonical chemokine receptors (CXCR4 (left) and CCR7 (right), black lines) and mGPR182 (red lines) or hGPR182 (green lines). CaCl_2_ + Ionomycin were added for comparison. (N = average of 15 cells, performed in duplicate at least twice). **(B)** ERK phosphorylation was measured by western blot (upper panel) upon stimulation with 50 nM CXCL11 (X11) or CXCL12 (X12) using 300.19 pre B cells expressing CXCR3, CXCR4, mGPR182 or hGPR182 (from left to right). Bar charts show quantification of normalized (loading control) pERK signal intensity from western blots (relative to unstimulated controls). N = 2 independent experiments performed in duplicates. Error bars ± SD. Dashed line represents threshold. Unpaired two-tailed t-test *p<0.05 **p<0.01. **(C–F)** Analyses of chemokine-induced responses in HeLa cells expressing mGPR182 **(C, E)** and hGPR182 **(D, F)**. Recruitment of LgBIT-miniG_αi_ to mGPR182-nLuc11 **(C)** or hGPR182-nLuc11 **(D)** and G protein activation measured as dissociation of Gα-nLuc from Gβγ-cpVenus in cells expressing mGPR182 **(E)** or hGPR182 **(F)** stimulated with 300 nM chemokine. HeLa cells expressing hCCR6-nLuc11 (black dots) served as positive controls upon stimulation with hCCL20. (N = 3, mean ± SEM).

Next, we investigated whether, upon ligand binding, GPR182 signals through typical chemokine receptor-induced pathways. We first tested chemokine-induced raises in intracellular free calcium. Fura-2-loaded 300.19 cells expressing human or mouse GPR182 (or hCXCR4 and hCCR7 as controls) were stimulated with hCXCL12 (**Figure 3A**, left) or hCCL19 (**Figure 3A**, right). Neither chemokine was able to induce intracellular calcium flux in cells expressing human or mouse GPR182, whereas cells expressing the cognate receptors for hCXCL12 and hCCL19, hCXCR4 or hCCR7, respectively, responded as expected. Next, we measured MAPK cascade activation upon addition of chemokines to cells expressing hCXCR3 (stimulating with hCXCL11), hCXCR4 (stimulating with hCXCL12), mGPR182 and hGPR182. While CXCL11 and CXCL12 induced ERK phosphorylation in CXCR3- and CXCR4-expressing cells, respectively, cells expressing mouse or human GPR182 did not respond to stimulation (**Figure 3B**).

Upon stimulation, canonical chemokine receptors can signal through Gα_i_ (60). We therefore investigated whether GPR182 can recruit mini Gα_i_-protein upon ligand stimulation using a split luciferase luminescence assay (47, 61). To this end, HeLa cells were co-transfected with human or mouse GPR182 or hCCR6 tagged with nLuc11 and the LgBIT-miniGα_i_ plasmid. While control cells expressing hCCR6 showed strong luminescence signal resulting from recombined luciferase when stimulated with the cognate ligand hCCL20, cells expressing either mGPR182 (**Figure 3C**) or hGPR182 (**Figure 3D**) failed to do so. Similarly, we measured heterotrimeric G_-_protein activation with a BRET assay (23). We expressed pIRES Gα_i_-nLuc Gβγ-cpVenus in HeLa cells and measured ligand induced dissociation of Gα-Gβγ which causes the reduction of the BRET signal. While hCCL20 induced G protein activation in CCR6-expressing cells, none of the tested chemokines induced this in either mGPR182-expressing (**Figure 3E**) or hGPR182-expressing cells (**Figure 3F**).

**Figure 4:**
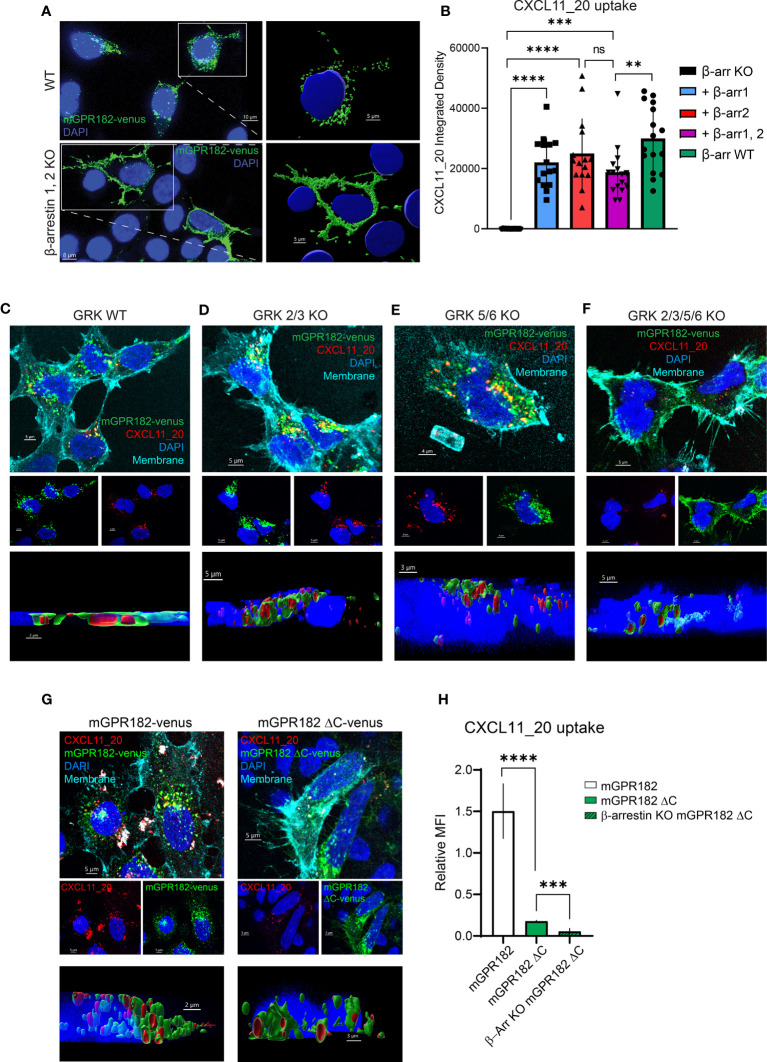
GPR182 requires GRK and β-arrestins for constitutive internalization and scavenging, but not its C-terminus. **(A)** Confocal images illustrating expression of tagged mGPR182-venus (green) in HEK293 WT cells (upper panels) or in HEK293 cells lacking β-arrestin expression (β-arrestin 1,2 KO) (lower panels), with enlargements (right) showing surface rendering of endosomal-like structures in WT cells and of receptor surface expression in KO cells (nuclei in blue, DAPI). Scale bars = 5 to 10 μm. **(B)** Quantification of integrated fluorescence intensities measured by confocal microscopy (measured with ImageJ) of internalized CXCL11_20-ATTO565 (200 nM) in HEK293 cells expressing mGPR182-venus. From left: cells deficient for β-arrestins (KO black column), cells expressing β-arrestin 1 (squares, blue column), β-arrestin 2 (upward triangle, red column), combined β-arrestin 1 and 2 (downward triangles, purple column), and HEK293^WT^ (black circles, green column). Data from one representative experiment, 3 fields of view were analyzed with at least 5 cells per field of view measured. Error bars ± SD. Unpaired two-tailed t-test, **p<0.01 ***p<0.001 ****p<0.0001. **(C–F)** Confocal images illustrating expression of mGPR182-venus (green), CXCL11_20 uptake (red), plasma membrane staining (cyan) and DAPI nuclear staining (blue) in HEK293 WT **(C)**, GRK 2/3 KO **(D)**, GRK 5/6 KO **(E)**, and GRK 2/3/5/6 KO cells **(F)**. Middle panels show DAPI with mGPR182-venus (blue/green), or DAPI with CXCL11_20 (blue/red). Bottom panels depict 3D rendering showing chemokine internalization in endosomal like structures. Scale bars = 5μm **(C, D, F)** and 4μm **(E)**. **(G)** Confocal images showing expression of mGPR182-venus (green, left), or mGPR182ΔC-venus (green, right), CXCL11_20 uptake (red), plasma membrane staining (cyan) and DAPI nuclear staining (blue) in HEK293 cells. Middle panels show DAPI with mGPR182-venus (blue/green), or DAPI with CXCL11_20 (blue/red). Bottom panels depict 3D rendering showing chemokine internalization in endosomal like structures. Scale bars = 5μm (top and middle panels), 2μm (bottom, left) 3μm (bottom, right). **(H)** MFI of CXCL11_20 uptake relative to the fluorescence of Venus measured by FACS in HEK293^WT^ cells expressing GPR182-Venus (white bar) or mGPR182ΔC-venus (green bar), β-arrestin 1,2^KO^ cells expressing mGPR182ΔC-venus (β-Arr KO mGPR182ΔC, hatched green bar). MFI of surface bound CXCL11_20 was measured by incubating cells with chemokine at 17°C and was subtracted from MFI of uptake at 37°C. N = 3. Error bars ± SD. Unpaired two-tailed t-test, ***p<0.001 ****p<0.0001.

β-arrestin 2 was previously shown to be necessary for ligand-independent internalizationof GPR182 (19, 20). Here we investigated the necessity for both β-arrestins 1 and 2 in GPR182 internalization and chemokine scavenging. We expressed mouse or human GPR182 tagged at the C-terminus with the fluorescent protein Venus (mGPR182-venus or hGPR182-venus) to explore its subcellular localization in wild type (WT) HEK293 or in HEK293 cells lacking both, β-arrestin 1 and β-arrestin 2 (β-arrestins 1, 2^KO^). While even in the absence of ligand GPR182 spontaneously localized in intracellular endosomal structures in HEK293^WT^ cells (**Figure 4A** top panel, S5A-C left panels), in β-arrestins 1, 2^KO^ cells it was exclusively found at the plasma membrane, unable to spontaneously internalize (**Figure 4A** bottom panel, **Figures S5A–C** right panels). To determine the necessity of both β-arrestins 1 and 2 for receptor trafficking, we performed rescue experiments by transfecting separately either β-arrestin 1 or 2 into HEK293 β-arrestins 1, 2^KO^ cells. We previously generated a GPR182-specific chimeric chemokine (30), CXCL11_20, that can be used to monitor scavenging GPR182 activity. The chimera is composed of the N-terminus of hCXCL11 and the main body of mCCL20 and was shown to selectively bind to GPR182 (30). In the absence of β-arrestins chemokine uptake was completely abolished (**Figure 4B**, **Figure S6A**). To determine whether receptor internalization and chemokine uptake could be rescued by re-expression of β-arrestins, we incubated HEK293 β-arrestin 1,2^KO^ cells transfected with β-arrestins with CXCL11_20, and measured uptake. Both, β-arrestin 1 and β-arrestin 2 alone or in combination rescued chemokine scavenging (**Figure 4B**, **Figure S6A**). The data were consistent with the receptor trafficking shown in **Figure 4A**, indicating that either β-arrestin molecule is sufficient to induce proper GPR182 internalization and chemokine scavenging. To determine possible ligand-induced β-arrestin recruitment we used a split luciferase assay between receptors tagged with nLuc11 and β-arrestins with inserted nLuc156 for recombination. However, addition of various chemokines did not appear to induce an increase in the luminescence signal, suggesting no ligand-induced recruitment of β-arrestin 1 or 2 to either mGPR182 (**Figure S6B**) or hGPR182 (**Figure S6C**). Notably, untreated cells transfected with GPR182 showed a marked luminescence signal already in the absence of chemokines, whereas unstimulated hCCR6-expressing cells (controls) showed a low basal luminescence, which was markedly increased upon stimulation with hCCL20. The finding suggests constitutive ligand-independent GPR182 β-arrestin interaction. Based on the finding that β-arrestins are required for constitutive receptor internalization, we asked what the effect of knocking-out GPCR kinases (GRK) in cells expressing GPR182 could be. Knockout of GRK 2/3 had no effect on receptor internalization and chemokine uptake, while knockout of GRK 5/6 caused a significant reduction, yet not complete, of internalization and uptake (**Figures 4C–E**, **Figure S6D**). However, knockout of all four GRK 2/3/5/6 lead to receptor localization at the plasma membrane similarly to the phenotype of β-arrestin 1, 2^KO^ and markedly reduced (but not completely absent) chemokine uptake (**Figure 4F**, **Figure S6D**) suggesting an important contribution, but not exclusive, of GRKs for GPR182 internalization.

GRKs are thought to phosphorylate ACKRs at the C-terminus to recruit β-arrestins. We therefore generated human or mouse GPR182 with a truncated C-terminus fused to the fluorescent protein Venus (mGPR182 ΔC or hGPR182 ΔC) (**Figure S7A**). Interestingly, the truncated version was still able to internalize and scavenge some CXCL11_20 into intracellular endosomes, as clearly shown by the 3D reconstruction and FACS analysis (**Figures 4G, H**, **Figures S7B, C, E**). The observation suggests the existence of a GRK-independent and β-arrestin-dependent internalization pathway. To investigate the possibility that β-arrestin interacts with the second intracellular loop (62) of hGPR182, we generated a mutant in which all serine and threonine residues of the loop were substituted with alanines (hGPR182 ST/A, **Figure S7A**). However, this did not replicate the β-arrestin 1,2^KO^ phenotype, as the receptor was present in endosomes and chemokine uptake was observed to the same levels as hGPR182 WT (**Figures S7D, E**).

Furthermore, we generated chimeric receptors in which we fused the C-terminus of GPR182 to CXCR4, and vice-versa (the C-terminus of CXCR4 fused to GPR182). We found that the C-terminus of GPR182 could mediate CXCL12-stimulated recruitment of β-arrestin 2 to CXCR4 (**Figure S8A**). However, the C-terminus did not suffice to induce the high basal association of β-arrestin 2 to CXCR4 as seen for GPR182 (**Figure S8A**), further indicating that GPR182 interacts with β-arrestins in an unconventional way.

Together, these data suggest that GPR182 requires β-arrestins for trafficking and that the receptor apparently spontaneously interacts with β-arrestins. The observation further implicates that GPR182 acts as an atypical chemokine receptor.

### GPR182 is the scavenger for CXCL13 and CCL28

**Figure 5:**
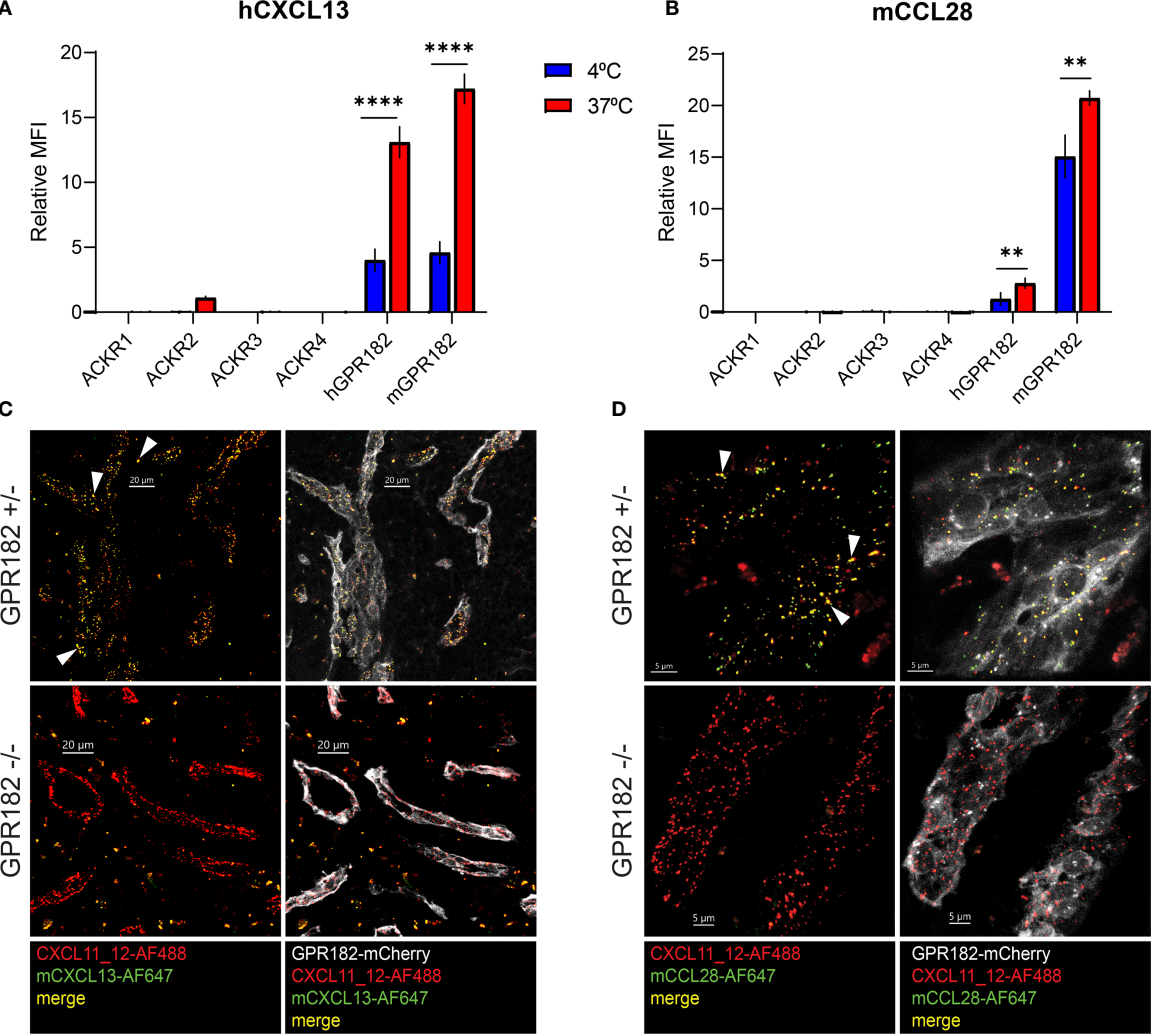
CXCL13 and CCL28 are GPR182-specific ligands. MFI of binding (4°C, blue columns) and uptake (37°C, red columns) of 100 nM hCXCL13 **(A)** and mCCL28 **(B)** by 300.19 pre-B cells expressing either ACKR1, ACKR2, ACKR3, ACKR4, mGPR182 or hGPR182 (T2A GFP). The background MFI of chemokines incubated with untransfected 300.19 pre-B cells was subtracted. Pooled data from at least 2 independent experiments, error bars ± SD. Unpaired two-tailed t-test, **p < 0.01, ****p < 0.0001. **(C, D)** Confocal images showing uptake in spleen sinusoids of mice co-injected i.v. (2.5 μM each) with fluorescently labelled mCXCL13-AF647 (green) **(C)** and CXCL11_12-AF488 (red) (scale bar = 20μm) or mCCL28-AF647 (green) **(D)** and CXCL11_12-AF488 (red) (scale bar = 5μm) into GPR182^+/-^ and GPR182^-/-^ mice (mCherry GPR182 reporter expression in grey). White arrows indicate areas of colocalization between red and green (yellow). Representative data from at least two independent experiments.

Most chemokines bind and are scavenged by one ACKR. However, few chemokines do not bind to the known ACKRs (1–4), including CXCL13 and CCL28. Recent studies highlight that CXCL13 is not a ligand for ACKR4 (10), contrary to previously thought (11). We have demonstrated that both chemokines could be bound and taken up by GPR182 *in vitro* (**Figure 1A**, **Figure S2**). To further confirm that GPR182 is their sole scavenger, we tested binding (at 4°C) and uptake (at 37°C) of hCXCL13 and mCCL28 by all known ACKRs *in vitro*. Indeed, no ACKR was able to effectively bind or take up excess (100 nM) amounts of either chemokine, with the exception of hGPR182 and mGPR182 (**Figures 5A, B**).

In order to study these interactions in more detail, we used a previously generated GPR182^mCherry^ knock-in reporter and knockout (KO) mouse (30). In hemizygous mice, the mCherry coding region replaces one allele of GPR182, resulting in a reporter mouse that can still express GPR182 from the intact allele. In homozygous mice, both alleles are replaced by the mCherry reporter, effectively generating a GPR182 knockout (KO) phenotype. We i.v. injected fluorescently labelled mouse CXCL13 (**Figure 5C**) and CCL28 (**Figure 5D**) into healthy young GPR182 heterozygous (GPR182^+/-^) and knockout (GPR182^-/-^) mice, and analyzed the localization of internalized chemokines in spleen endothelial cells, where GPR182 is highly expressed (30, 31). As positive control, we co-injected CXCL11_12, a chimeric chemokine that binds both GPR182 and ACKR3, as ACKR3 is expressed in the same endothelial cells (30, 49). While in GPR182^+/-^ mice we could observe endosomes containing either both CXCL13 and CXCL11_12 (yellow) (**Figure 5C**) or both CCL28 and CXCL11_12 (yellow) (**Figure 5D**), GPR182^-/-^ mice only possessed CXCL11_12^+^ endosomes (red) indicating that no CCL28 or CXCL13 (green) were taken up (**Figures 5C, D**). Together, the data strongly suggests that GPR182 is the sole scavenger for CCL28 and CXCL13 and no other ACKR internalizes these chemokines *in vivo*.

### Chemokine levels are elevated in healthy GPR182^-/-^ mice

**Figure 6:**
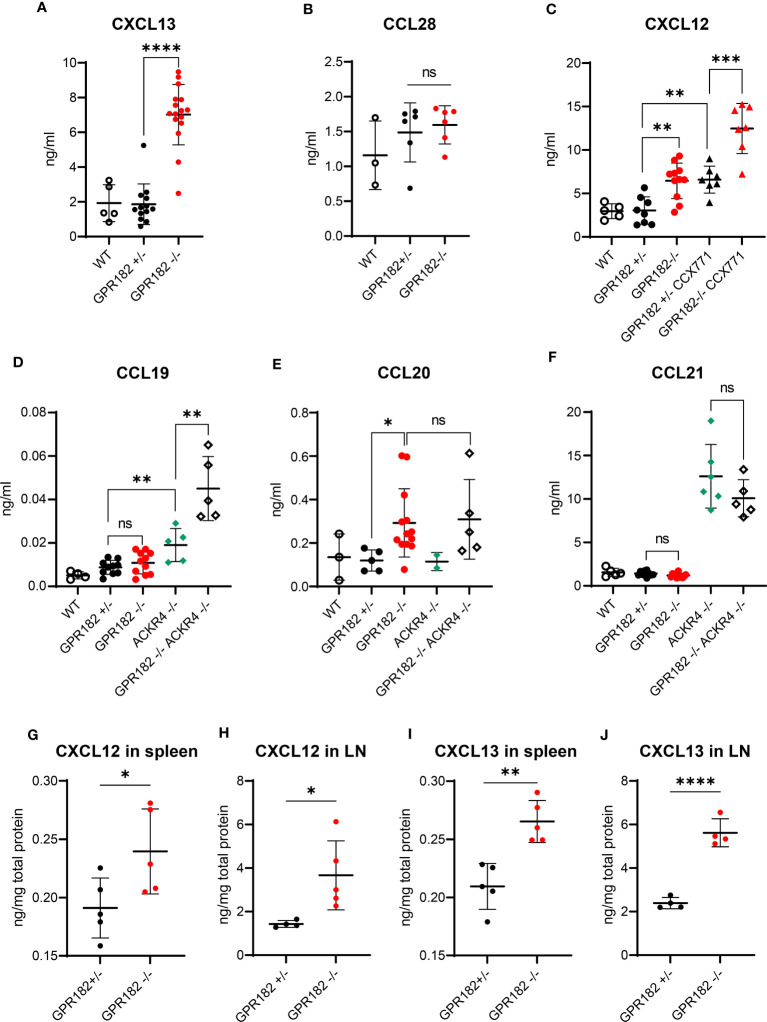
GPR182 regulates serum chemokine levels. **(A)** Serum levels of CXCL13 in C57B6 (open circles), GPR182^+/-^ (black) and GPR182^-/-^ (red) mice. **(B)** Serum level of CCL28 in C57B6 (open circles), GPR182^+/-^ (black) and GPR182^-/-^ (red) animals. **(C)** Serum levels of CXCL12 in C57B6 (open circles), GPR182^+/-^ (black circles), GPR182^-/-^ (red circles), GPR182^+/-^ treated with CCX771 (black triangles), and GPR182^-/-^ treated with CCX771 (red triangles) animals. Serum level of CCL19 **(D)**, CCL20 **(E)**, CCL21 **(F)** in C57B6 (open circles), GPR182^+/-^ (black circles), GPR182^-/-^ (red circles), ACKR4^-/-^ (green diamonds), GPR182^-/–^ACKR4^-/-^ (open diamonds) animals. **(G–J)** Interstitial chemokine levels: CXCL12 **(G)** in spleen and **(H)** in LN, CXCL13 **(I)** in spleen and **(J)** in LN. Chemokine levels were normalized to total protein content. (N=3-16 per group. Error bars means ± SD (means thick horizontal line). Unpaired two-tailed t-test *p<0.05, **p<0.01, ***p<0.001, ****p<0.0001, ns not significant).

To determine whether GPR182 scavenging activity regulates chemokine levels *in vivo*, we first analyzed serum chemokine concentrations in wildtype (WT), GPR182^+/-^ and GPR182^-/-^ mice. While CXCL13 serum levels remained unchanged in GPR182^+/-^ compared to WT, GPR182^-/-^ mice displayed a 3.5-fold increase (**Figure 6A**). This suggests that just one copy of the GPR182 gene suffices for receptor function. Interestingly, deletion of GPR182 had no effect on the serum levels of CCL28 (**Figure 6B**).

CXCL12 levels were also unvaried in GPR182^+/-^, but increased 2-fold in GPR182^-/-^ mice (**Figure 6C**). As CXCL12 is also scavenged by ACKR3, we sought to determine whether ACKR3 and GPR182 could cooperate in regulating CXCL12 levels. We treated GPR182^+/-^ and GPR182^-/-^ with the ACKR3 competitive agonist CCX771 (63) and collected blood 2h later. The time of treatment was sufficient to elevate CXCL12 serum levels 2-fold in CCX771 treated GPR182^+/-^ compared to untreated animals in accordance with previous reports (36, 38). However, inhibition of ACKR3 in GPR182^-/-^ animals resulted in a striking almost 4-fold apparent additive increase in CXCL12 levels (**Figure 6C**).

We also measured serum levels of ACKR4 ligands, including CCL19, CCL20 and CCL21. To determine the cooperation of ACKR4 and GPR182 in regulating these chemokines we crossed GPR182^-/-^ to ACKR4^-/-^ mice (55), generating GPR182-ACKR4 double KO animals (DKO). In the serum, an increase in CCL19 was only observed in ACKR4 single KO, and not in GPR182 single KO (**Figure 6D**). However, DKO mice displayed an even further increase in CCL19 serum levels, suggesting a relationship between ACKR4 and GPR182 in regulating CCL19 levels. CCL20, which binds GPR182 with moderate affinity (**Table 1**), is a recently identified ACKR4 ligand (10, 12). However, we were unable to detect an increase of CCL20 in the serum of ACKR4^-/-^ mice (**Figure 6E**). Nonetheless, GPR182^-/-^ displayed elevated CCL20 levels, reflecting its higher affinity (120 nM), compared to that of ACKR4 for CCL20 (800 nM) (12). DKO had no further effect on CCL20 levels compared to GPR182^-/-^ (**Figure 6E**). CCL21, which binds GPR182 with extremely low affinity (precluding us from being able to measure its affinity by competition binding experiments *in vitro*), was only elevated in ACKR4^-/-^ mice, with DKO displaying no difference compared to ACKR4 single KO (**Figure 6F**).

We and others previously showed that GPR182 is broadly expressed in the vasculature of multiple organs, predominantly in spleen sinusoids and lymph node (LN) lymphatic vasculature and high endothelial venules (HEV) (19, 30, 31). We therefore measured chemokine levels in the interstitium of these organs. We found at least a 2-fold increase of both CXCL12 and CXCL13 levels in both spleen and lymph nodes of GPR182^-/-^ compared to GPR182^+/-^ mice (**Figures 6G–J**). However, no significant differences were seen in CCL19, and CCL21 organ content (**Figure S9A–D**). CCL20 in organs was below the detection limit, therefore could not be measured. We also previously showed that GPR182 is expressed in intestine and colon (30). CCL28, which is a mucosal chemokine, levels were slightly yet not significantly elevated in fecal supernatants (**Figure S9E**).

Taken together, these data suggest that GPR182 is able to scavenge chemokines and remove them from the circulation or interstitium. It is primarily involved in clearing CXCL13 and CXCL12 from serum and SLO, with moderate effects on CCL20 and CCL19 levels in the serum. Moreover, GPR182 cooperates with ACKR3 and ACKR4 in regulating CXCL12 and CCL19 serum levels, respectively.

### GPR182^-/-^ mice have a reduced marginal zone, and a reduced ability to respond to T-independent antigens

**Figure 7:**
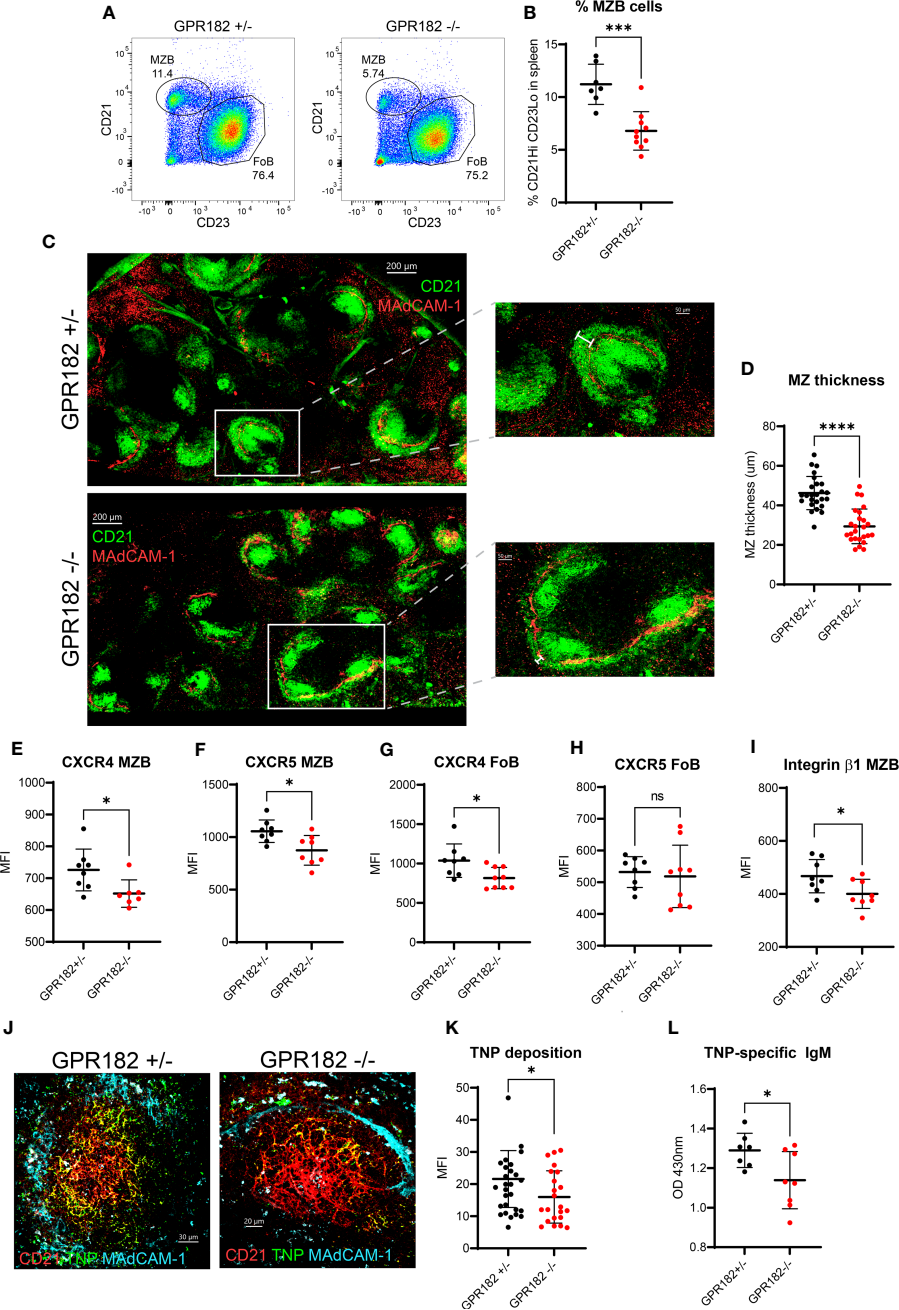
GPR182^-/-^ mice have a reduced marginal zone. **(A)** Representative FACS plots indicating the percentage of MZBs (CD21^Hi^ CD23^-^ cells gated on CD19^+^ B220^+^) in GPR182^+/-^ (left) and GPR182^-/-^ (right) mice. **(B)** Quantification of multiple measurements, means ± SD (N = at least 7/group). **(C)** Representative confocal images of CD21/35 staining (green) to show MZ, found at the border with the marginal sinus, MAdCAM-1 (red) (left, scale bar = 200μm). Right panels: enlargements with measurement bar used for quantification (right, scale bar = 50μm). **(D)** Quantification of MZ thickness (N=5 spleens/group, with at least 5 follicles/spleen analyzed). Mean fluorescence intensity (MFI) of receptor expression on MZBs CXCR4 **(E)** and CXCR5 **(F)**; and on FoBs CXCR4 **(G)** and CXCR5 **(H)**, CD29 expression (integrin β1 chain) on MZB **(I)** (N= at least 7 mice/group). **(J)** Representative confocal images of deposition of TNP-Ficoll-fluorescein (green) on spleen FDCs (CD21, red) within the follicle (lined by MAdCAM-1^+^ marginal sinus, cyan), in GPR182^+/-^ (left) and GPR182^-/-^ (right) mice, 3h post-injection. **(K)** Quantification of TNP-Ficoll-fluorescein deposition on CD21^+^ FDCs of GPR182^+/-^ (black circles) and GPR182^-/-^ (red circles) mice (N=5 mice/group). **(L)** Levels of TNP-specific IgM at day 4 post immunization in serum of GPR182^+/-^ (black circles) and GPR182^-/-^ (red circles) mice measured by ELISA (N=7 mice/group). Error bars means ± SD, mean thick horizontal line. Unpaired two-tailed t-test: *p<0.05, ***p<0.001, ****p<0.0001, ns not significant).

We found no haploinsufficiency in GPR182^+/-^ mice indicating that the hemizygous reporter is physiologically equivalent to the WT. We confirmed the phenotype detected by Le Mercier et al., in which GPR182^-/-^ mice display an enlarged spleen (**Figure S10A**) (19). Upon further analysis, we found that the percentage of total B cells, in particular follicular B cells (FoBs) remained constant between GPR182^+/-^ and GPR182^-/-^ (**Figures S10B, C**). However, we further explored the immune landscape of GPR182^-/-^ animals and observed that GPR182^-/-^ animals have an increased basal serum IgM level (**Figure S10D**), but not in IgG (**Figure S10E**). This was accompanied by an increase in the number of plasma cells in the spleen (**Figure S10F**), and more importantly in peritoneal B1 (**Figure S10G**) and particularly B1a B cells (**Figure S10H**), which are known to be source of natural IgM. Moreover, we observed by flow cytometry that GPR182^-/-^ mice have a lower percentage of marginal zone B cells (MZBs) (**Figures 7A, B**). In GPR182^-/-^, this was accompanied by a thinner marginal zone (MZ) measured by confocal imaging (**Figures 7C, D**). Furthermore, both MZBs and FoBs presented reduced levels of surface CXCR4, and MZBs also CXCR5 (**Figures 7E–H**), along with the reduction of the integrin β1 chain (CD29) (**Figure 7I**). The MZ is necessary to provide an early humoral response to type II T-independent antigens, such as TNP-Ficoll. Because of the defect in MZ size and cellularity, we sought to investigate the humoral response of GPR182^-/-^ mice to this antigen. Indeed, we found that a reduced amount of fluorescently labelled T-independent antigen, TNP-Ficoll-fluorescein, was deposited on CD21^+^ follicular dendritic cells (FDCs) (**Figures 7J, K**). Furthermore, we found that 4 days after immunization, anti-TNP IgM levels were reduced in the serum of GPR182^-/-^ animals compared to heterozygous counterparts (**Figure 7L**). Furthermore, the increased CXCL12 and CXCL13 in the serum and SLO, and reduced ability to respond to T-independent antigens, prompted us to investigate the germinal center response to T-dependent antigens in GPR182^-/-^ mice. However, 14d after NP-KLH immunization, we did not observe differences in magnitude of antibody response (**Figures S11A, B**), either high affinity (left, NP4) or low affinity (right, NP20), and neither in GC B cells (**Figure S11C**) or T follicular helper (Tfh) (**Figures S11D, E**) percentages. Overall, the data suggests that GPR182 is necessary to scavenge chemokines to ensure correct architecture, development and function of the MZ, which in turn allows for appropriate responses to cognate antigens; and that GPR182 expressed on stromal cells might be involved in the homing of peritoneal B cells (64), but not in the response to T-dependent antigens.

## Discussion

Atypical chemokine receptors predominantly function as scavengers removing chemokines from the circulation and from local environments. The latter activity contributes to the fine-tuning of attractant gradients necessary for leukocyte migration and maintaining SLO architecture. In addition to binding CXCL9, CXCL10, CXCL12 and CXCL13 (19, 20), we extend the binding and uptake profile of GPR182 to multiple chemokines, albeit with low affinity, suggesting it is a broad chemokine scavenger.

In contrast to a previous report, we found CCL28 as selective ligand for the mouse and human scavenger GPR182 *in vitro* (19). However, at difference to other chemokines we did not detect elevated levels of CCL28 in the serum of GPR182^-/-^ healthy young mice. Despite the known expression of CCL28 in the intestine (65), we could also not find significant alterations of CCL28 in the feces, probably due to the low sensitivity of the detection system. It is conceivable that under inflammatory conditions possible elevations of interstitial levels in the mucosa are maintained by GPR182.

While most chemokines were shown to interact with one of the known ACKRs (10) binding and scavenging of CXCL13 and CCL28 appears to be unique to GPR182. We demonstrated here that indeed CXCL13 and CCL28 fail to bind to any other known ACKR *in vitro* and are not taken up by endothelial cells in GPR182^-/-^ mice *in vivo*. Moreover, CXCL13, which binds GPR182 with high affinity, is found highly elevated in the serum of GPR182^-/-^ mice. We observed that GPR182-proficient mice readily take up CCL28 and CXCL13 in endosomes of splenic sinusoids, while GPR182^-/-^ mice were unable to do so. We also directly show that GPR182 removes CXCL13 from *in vitro/ex vivo* cell culture supernatants, and degrades the chemokine through pH-sensitive endolysosomal pathways. Taken together, our study demonstrates that GPR182 is the ACKR for CXCL13 and CCL28.

In agreement with a previous report (20) we found that CXCL1 was not effectively taken up. Furthermore, we observed that hGPR182 has an overall reduced breadth and reduced affinities for chemokine, when compared to mGPR182. By contrast to Torphy et al., we found CXCL12 to have a slightly higher affinity for hGPR182 compared to hCCL20 (20). They also found CCL21 to bind effectively to hGPR182, while we only found minimal binding despite the chemokine being readily taken up by ACKR4 in control experiments. Moreover, we found that mGPR182 bound human and mouse CCL21, although to a lower degree compared to other chemokines.

GPR182 does not partake in canonical chemokine receptor signaling such as G-protein activation, calcium mobilization and MAPK activation. Although we found that chemokines do not induce the recruitment of β-arrestins to GPR182, the presence of either β-arrestin 1 or β-arrestin 2 were necessary for spontaneous receptor internalization and chemokine scavenging. This distinguishes GPR182 from the scavengers ACKR3 and ACKR4 for which β-arrestins are dispensable (23, 26, 27). Moreover, cooperation between GRK 2/3/5/6 is required for optimal internalization and scavenging (66). GRK 2/3 are known to be recruited to the plasma membrane by the βγ-subunits of activated heterotrimeric G-proteins (67). In the case of GPR182, which does not activate G-proteins, alternative pathways must be involved, similarly as described for ACKR3 (68, 69) and ACKR4 (23). Furthermore, we found that the C-terminus is in part dispensable for β-arrestin recruitment, receptor internalization and scavenging. This was not due to a non-functional C-terminus, but rather suggests an unconventional mode of interaction between GPR182 and β-arrestins that mainly rely on other receptor intracellular determinants than the C-terminus.

We previously described the expression pattern of GPR182 in the endothelium of multiple organs, including spleen sinusoids, lymph node high endothelial venules and lymphatic endothelium and liver sinusoids, which resembles the expression of other ACKRs (30). Indeed, this anatomical location allows immediate access to the bloodstream, which allows ACKRs, e.g. ACKR3, to bind and remove excessive chemokines from the circulation (36). Indeed, we noticed a marked elevation of CXCL13 and CXCL12 in both, serum and the interstitium of SLOs in GPR182-deficient animals. Moreover, we showed that GPR182 may collaborate with ACKR3 and partly with ACKR4 to regulate serum levels of CXCL12 and CCL19, respectively in healthy young mice. It is plausible that under inflammatory conditions where these homeostatic chemokines might be upregulated the role of GPR182 becomes more prominent. However, we did not find significantly elevated levels of CCL19 and CCL21 in the serum or SLO of GPR182^-/-^ mice, which may be attributed to the low affinity of the chemokines for GPR182 measured *in vitro*. By contrast, ACKR4^-/-^ animals clearly displayed increased levels of CCL19 and CCL21 in the serum. CCL19 levels were exacerbated in animals lacking both, ACKR4 and GPR182 suggesting that ACKR4 is the primary scavenger for CCL19 while GPR182 acts as backup. Hence, ACKR4 can fully compensate for the absence of GPR182 and prevent excessive increases of CCL19 in the serum. Moreover, when GPR182 is present but ACKR4 absent, GPR182 is able to maintain CCL19 levels to a relatively low level, although significantly increased compared to WT situations. By contrast to CCL19, the elevated levels of CCL21 in ACKR4^-/-^ mice were not further increased in DKO animals.

CCL20 is an inflammatory chemokine, which is upregulated during inflammation to recruit primarily CCR6-expressing leukocytes (70), with a well-established role in diseases such as multiple sclerosis or its animal model experimental autoimmune encephalomyelitis (71). Here we measured CCL20 serum levels at baseline, which often resulted in undetectable levels in naïve WT or heterozygous animals. CCL20 levels were also undetectable in SLO of naïve mice. Nevertheless, we identified CCL20 as ligand that is readily internalized *in vitro*, and ablation of GPR182 leads to elevated serum levels of the chemokine, suggesting its scavenging *in vivo* and having a critical role in CCL20 homeostasis. Our group and others had previously identified CCL20 as an ACKR4 ligand (10, 12). However, while GPR182^-/-^ mice showed increased CCL20 levels in the serum, ACKR4^-/-^ mice did not. In addition, DKO of GPR182 and ACKR4 did not further increase the levels of CCL20 measured in GPR182^-/-^ mice. The observation is consistent with the affinity of CCL20 for ACKR4 (around 800 nM (12)) and for GPR182 (around 120 nM). Of note, these experiments were performed under homeostatic conditions. We cannot exclude that this might change under inflammatory conditions.

Previous studies described in the absence of GPR182 mild phenotypes in zebrafish and mice in the hematopoietic stem cell compartment (19, 33). Here, we have identified a novel phenotype in GPR182^-/-^ mice. The animals displayed increased natural IgM, possibly derived from peritoneal B1a cells, which have been shown to rely on CXCL13 for homing and natural antibody production. Furthermore, these animals had a reduced marginal zone both, by cellularity and by size. We found reduced surface chemokine receptor levels in both MZBs and FoBs, suggesting desensitization and internalization of the receptors due to elevated cognate chemokine levels, which could impair their migration abilities during MZB homing and shuttling. This was also accompanied by reduced integrin β1 levels on MZBs, which is known to participate in anchoring MZBs to their niche (72, 73). Indeed, this prevents GPR182^-/-^ mice from efficiently responding to T-independent antigens, making GPR182 an immunomodulator of T-independent response. Interestingly, inhibition of ACKR3 or ablation of ACKR4 also results in perturbed MZ, highlighting the requirement for multiple chemokines and receptors in establishing and maintaining this delicate architecture (35, 37). While deletion of ACKR3 in B-cells prevented mice to build a MZ, GPR182, which is not expressed on MZBs, only reduces the architecture, which may explain the relatively small decrease in responses against TNP-Ficoll immunization compared to animals with B cell-specific ACKR3 deficiency (37). It also supports the finding that GPR182 is a broad but low affinity scavenger receptor.

CXCL13 and CXCL12 are both involved in germinal center organization promoting B cell cycling from light zone to dark zone. However, in GPR182^-/-^ animals where these chemokines are found to be elevated in both serum and SLOs, we could not detect marked changes in germinal center responses following NP-KLH immunization. This could suggest that the balance between chemokines rather than total chemokine levels is important for correct GC generation and function.

In conclusion, we identified GPR182 as broad chemokine binding receptor that scavenges chemokines *in vitro and in vivo*. Moreover, GPR182 binds and scavenges CXCL13 and CCL28, two chemokines which currently lack a paired ACKR. We could also link GPR182 to a novel immunological phenotype using GPR182^-/-^ mice. Overall, we demonstrate that GPR182 acts as a broadly scavenging atypical chemokine receptor, thus we propose the receptor to be named ACKR5. A schematic summary of our findings are depicted in **Figure S12**.

## Data availability statement

The original contributions presented in the study are included in the article/**Supplementary Material**. Further inquiries can be directed to the corresponding author.

## Ethics statement

The animal study was reviewed and approved by Swiss Federal Veterinary Office guidelines and authorized by the Animal Studies Committee of Cantonal Veterinary (License: 33/2020; 74/2022).

## Author contributions

Conceptualization: SM, MT. Methodology: SM, OG, MA, ST, PP, DL, MT. Investigation: SM, OG, MA, ER, GD’U, PA, ST, MT, AC, MS. Visualization: SM, OG, MA, DL, MT. Supervision: DL, MT. Writing—original draft: SM. Writing—review & editing: PA, MA, AC, GD’U, OG, AC, MS, PP, DL, SM, MT. All authors contributed to the article and approved the submitted version.
