# Supplementary material for: GPR182 is a broadly scavenging atypical chemokine receptor influencing T-independent immunity
Source: Front Immunol. 2023 Jul 24;14:1242531. doi: 10.3389/fimmu.2023.1242531 (PMC10405735; doi:10.3389/fimmu.2023.1242531)
Supplement: Supplementary file 1 [file DataSheet_1.pdf]

Supplementary material

Fig S1A

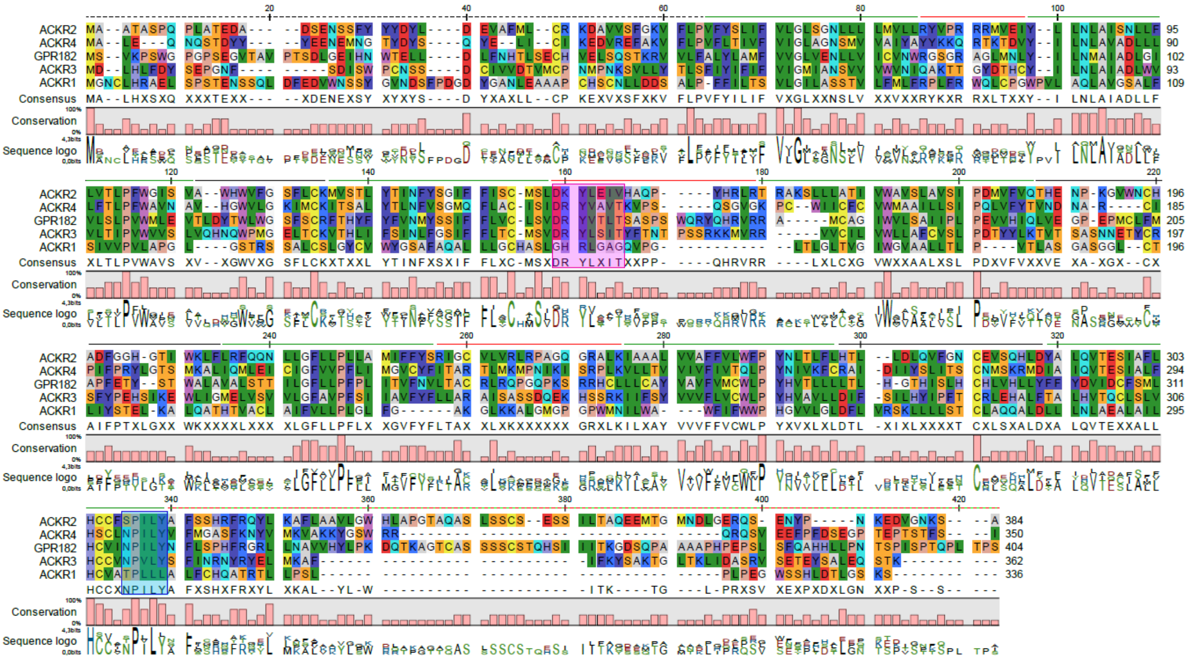

Fig S1B

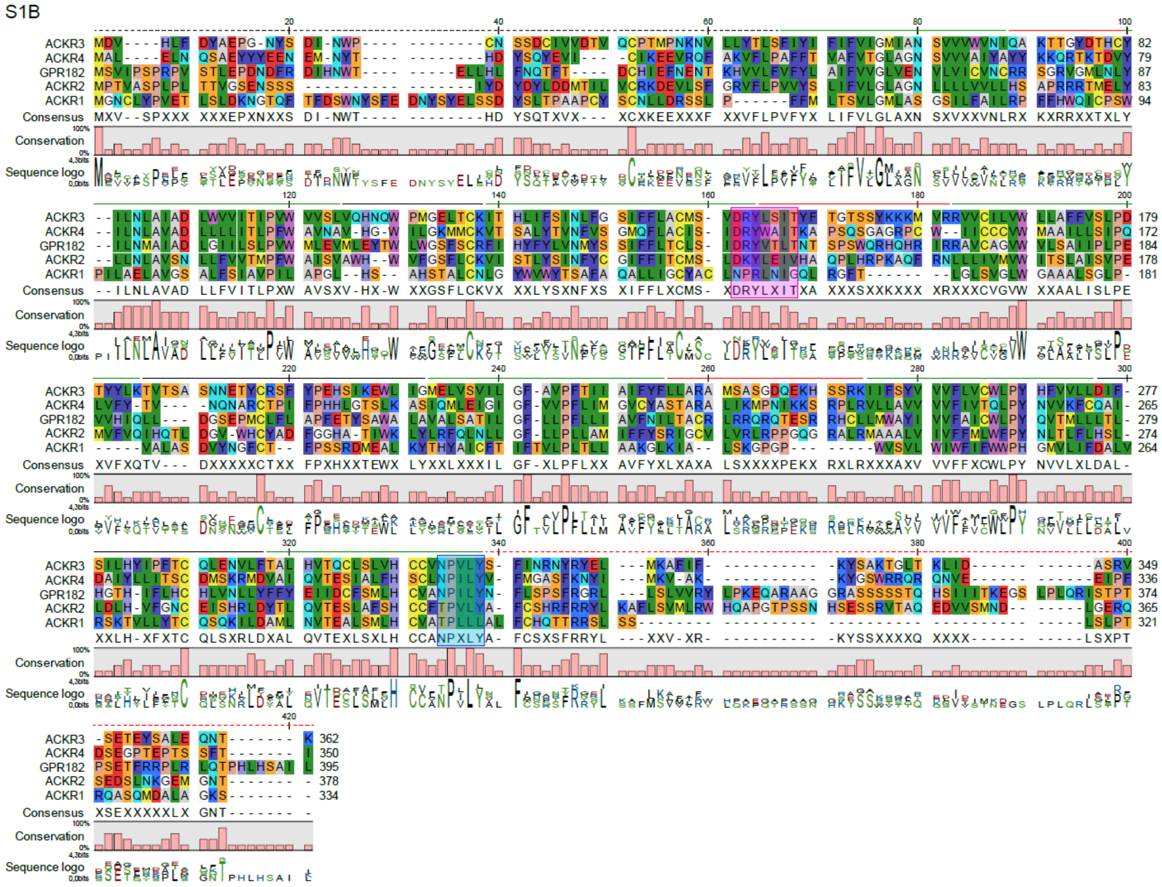

Fig S1C

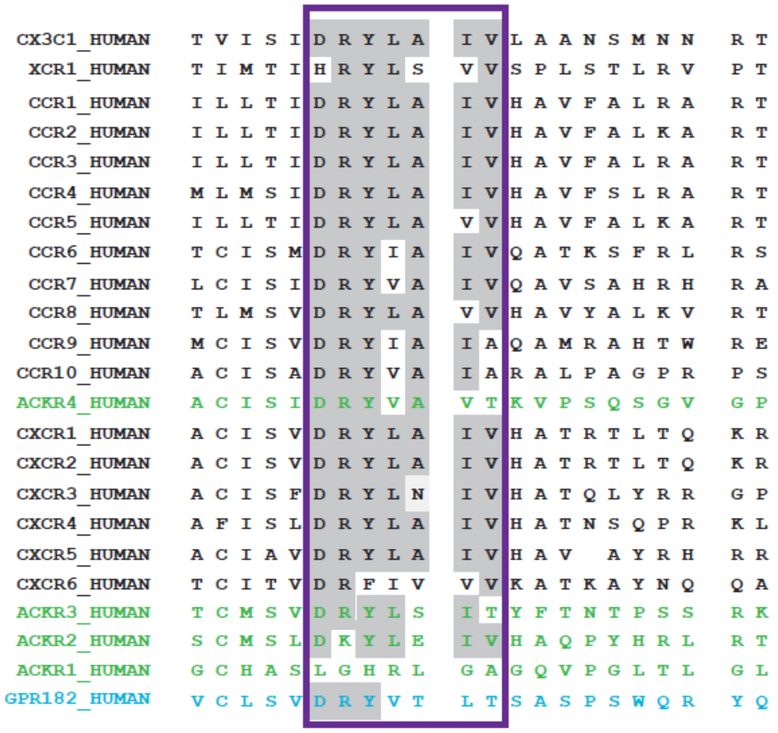

Fig S1D

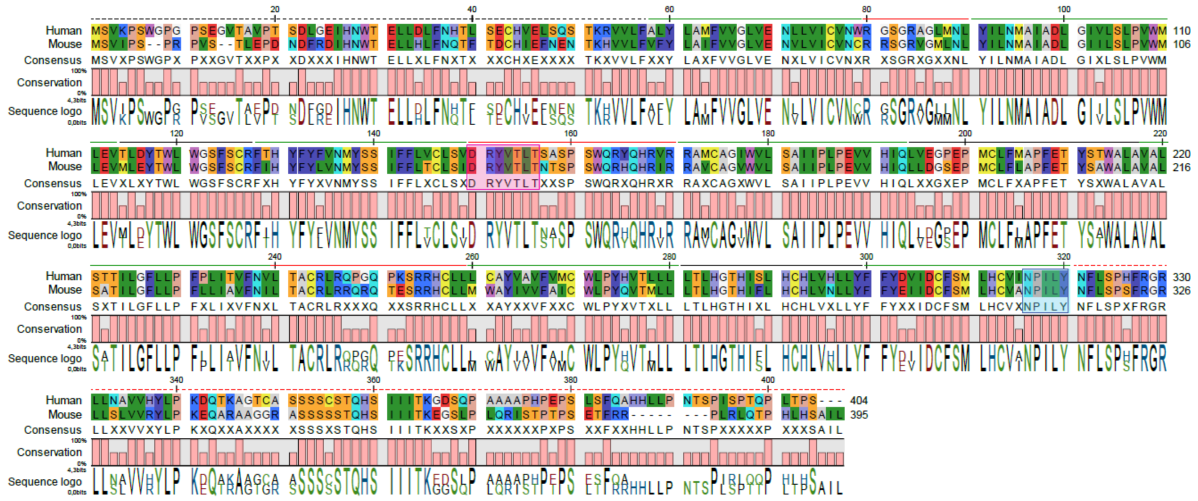

Fig S1. ACKR alignment. (A, B, D) Alignments of human (A) and mouse (B) ACKRs and GPR182, and human and mouse GPR182 (D). Black dotted line represents N-terminus, black lines represent extracellular loops green lines represent transmembrane regions, red lines represent intracellular loops, red dotted lines represent C-terminus (based on human GPR182 (A, D), mouse GPR182 (B)). The pink box highlights the region of the modified DRYLAIV motif, the cyan box highlights the region of NPxxY motif. **(C)** Alignment of human canonical (black) and atypical (green and cyan) chemokine receptors in the region surrounding the DRYLAIV motif. The purple box highlights the motif.

Figure S2

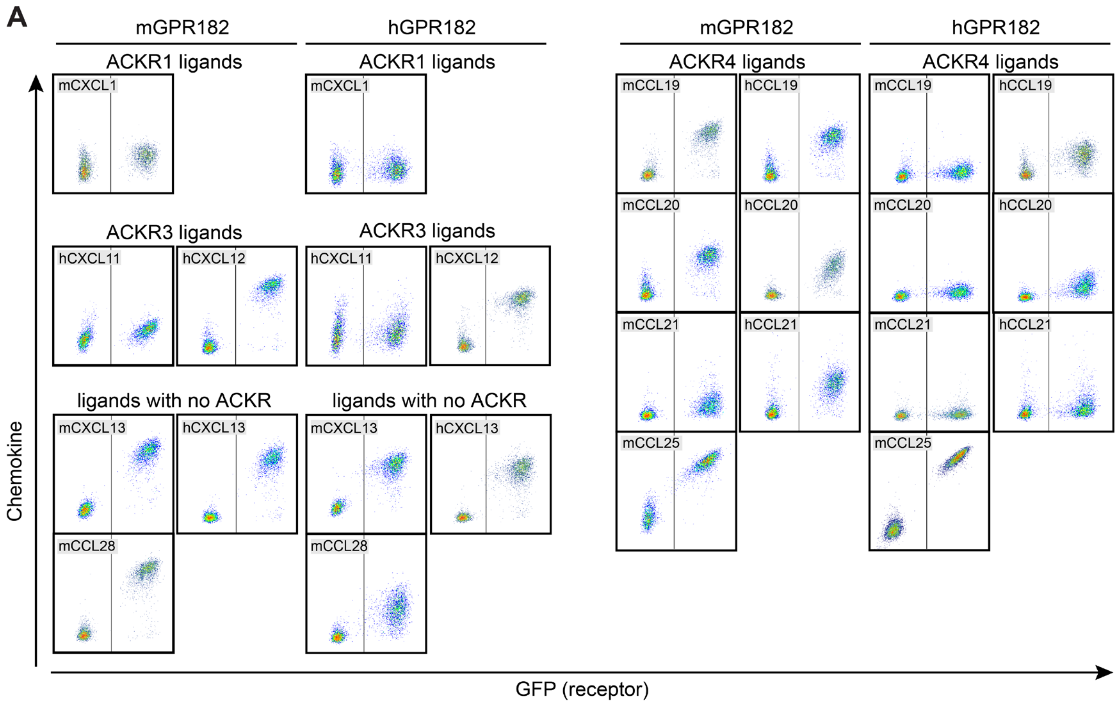

Fig S2. GPR182 internalizes a broad range of chemokines. (**A**) 300.19 pre-B cells expressing mGPR182 T2A GFP (left panels) or hGPR182 T2A GFP (right panels) were incubated (together with parental GFP negative 300.19 cells) with 20 nM fluorescently labelled human or mouse chemokines (as indicated in each panel) at 37ºC for 45 min and uptake was measured by FACS. Panels are grouped for shared binding and uptake by other ACKRs as indicated. Shown is one representative experiment out of 2 independent determinations performed.

Figure S3

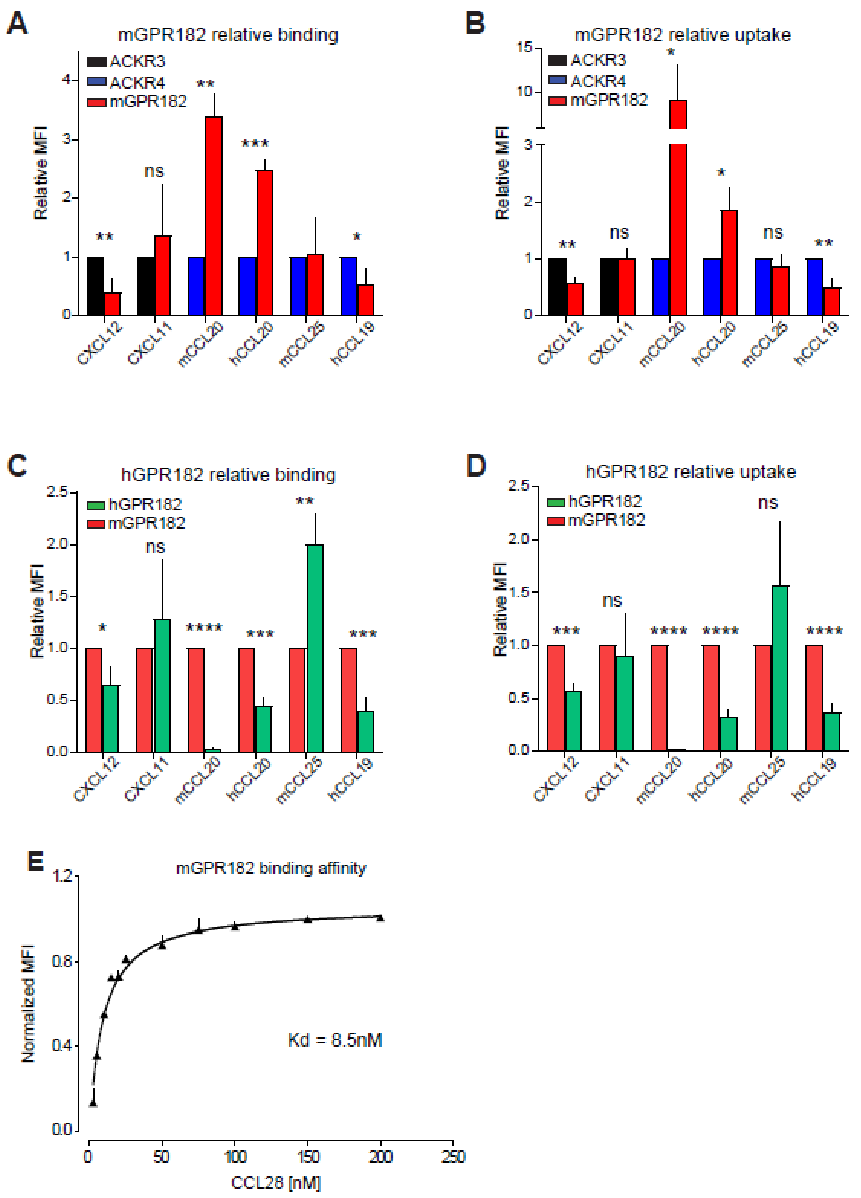

Fig S3. GPR182 displays lower binding and scavenging ability compared to most ACKRs. (**A**) mGPR182 binding or (**B**) uptake of indicated chemokines by mGPR182-expressing cells was measured by FACS. The MFI of fluorescent chemokine was normalized to GFP (T2A expressed) expression and compared to normalized MFI of known ACKRs (ACKR3 and ACKR4) for selected chemokines. Relative (**C**) binding or (**D**) uptake of hGPR182 compared to mGPR182. MFIs were normalized to receptor expression (GFP). N= at least 2 independent experiments. Error bars ± SD. Unpaired two-tailed t-test: *p<0.05, **p<0.01, ***p<0.001 ****p<0.0001, ns: not significant. (**E**) Increasing concentrations of fluorescently labelled CCL28-AF647 were incubated with 300.19 pre-B cells expressing mGPR182 and direct binding (4ºC) measured by FACS.

Figure S4

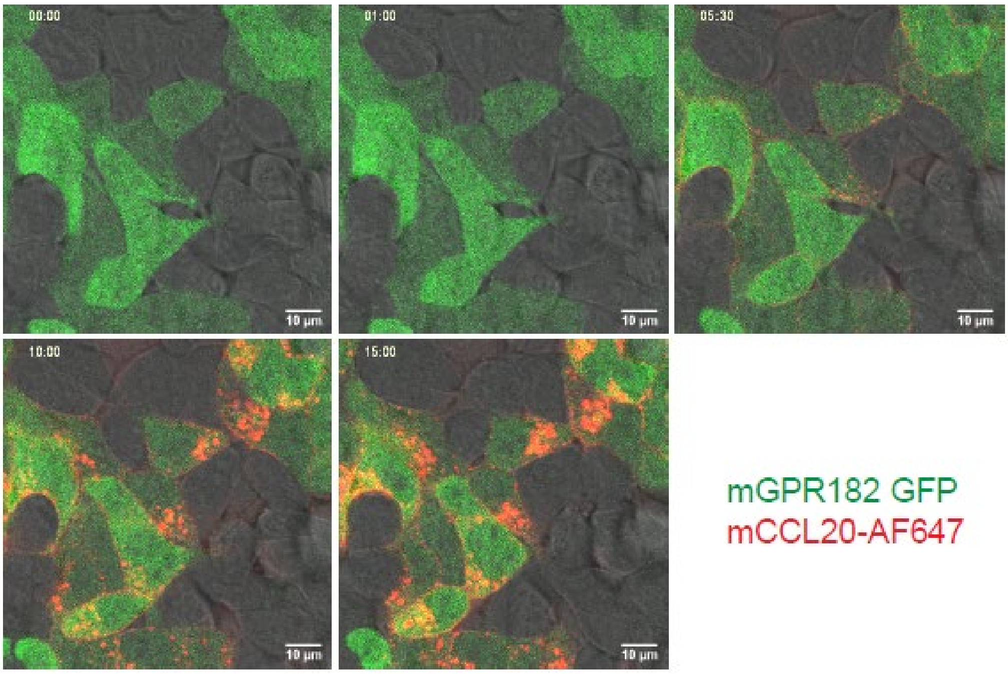

Fig S4 and Video S1. mCCL20-AF647 was added at t = 1 minute at a final concentration of 200 nM to HEK293 cells transiently transfected with mGPR182 T2A GFP. Endosomes containing CCL20 (red) appeared 4 minutes 30 seconds after the addition of the chemokine (5:30). (**Fig S2**) shows captured images from **Video S1** at t = 00:00 min (no chemokine added); t = 1 min; t = 5:30 min; t = 10 min; t = 15 min (scale bar = 10µm). Note only transfected cells (green) take up chemokine.

Figure S5

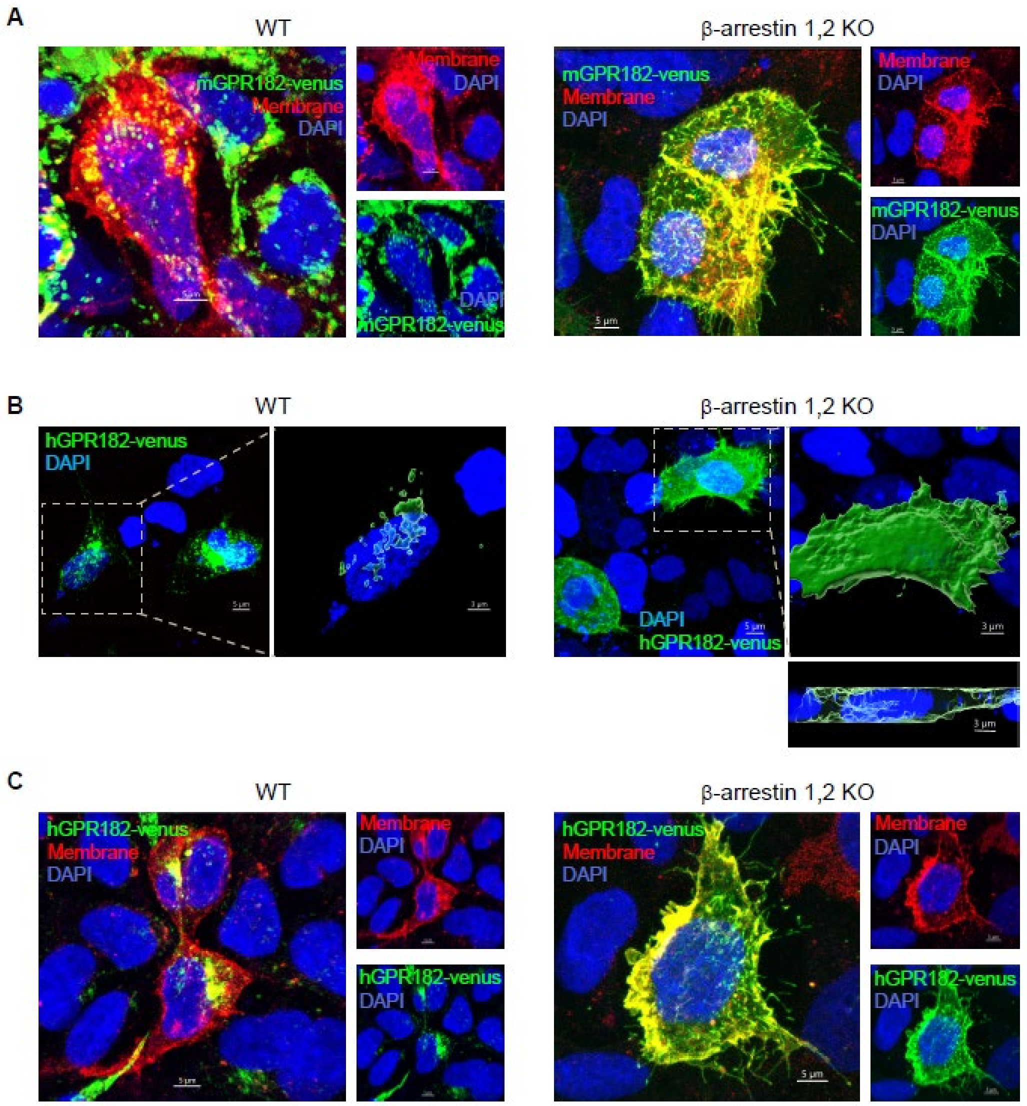

Fig S5. hGPR182 requires β-arrestins for constitutive internalization. (**A**) Confocal images illustrating expression of tagged mGPR182-venus (green) and plasma membrane labeling (Lck-mCherry, red) in $HEK293^{WT}$ cells (left panels) or in HEK293 cells lacking β-arrestin expression (β-arrestin 1,2 KO) (right panels). Scale bar = 5µm. Small panels show separate channels for GFP/DAPI and Lck-mCherry/DAPI. (**B**) Confocal images illustrating expression of tagged hGPR182-venus (green) in $HEK293^{WT}$ cells (left panels) or in HEK293 cells lacking β-arrestin expression (β-arrestin 1,2 KO) (right panels), with enlargements (right panels) showing surface rendering of endosomal-like structures in WT cells and of receptor surface expression in KO cells (nuclei in blue). Scale bars = 5µm, 3µm for enlargements. (**C**) Confocal images illustrating expression of tagged hGPR182-venus (green) and plasma membrane labeling (Lck-mCherry, red) in $HEK293^{WT}$ cells (left panels) or in HEK293 cells lacking β-arrestin expression (β-arrestin 1,2 KO) (right panels). Scale bar = 5µm.

Figure S6

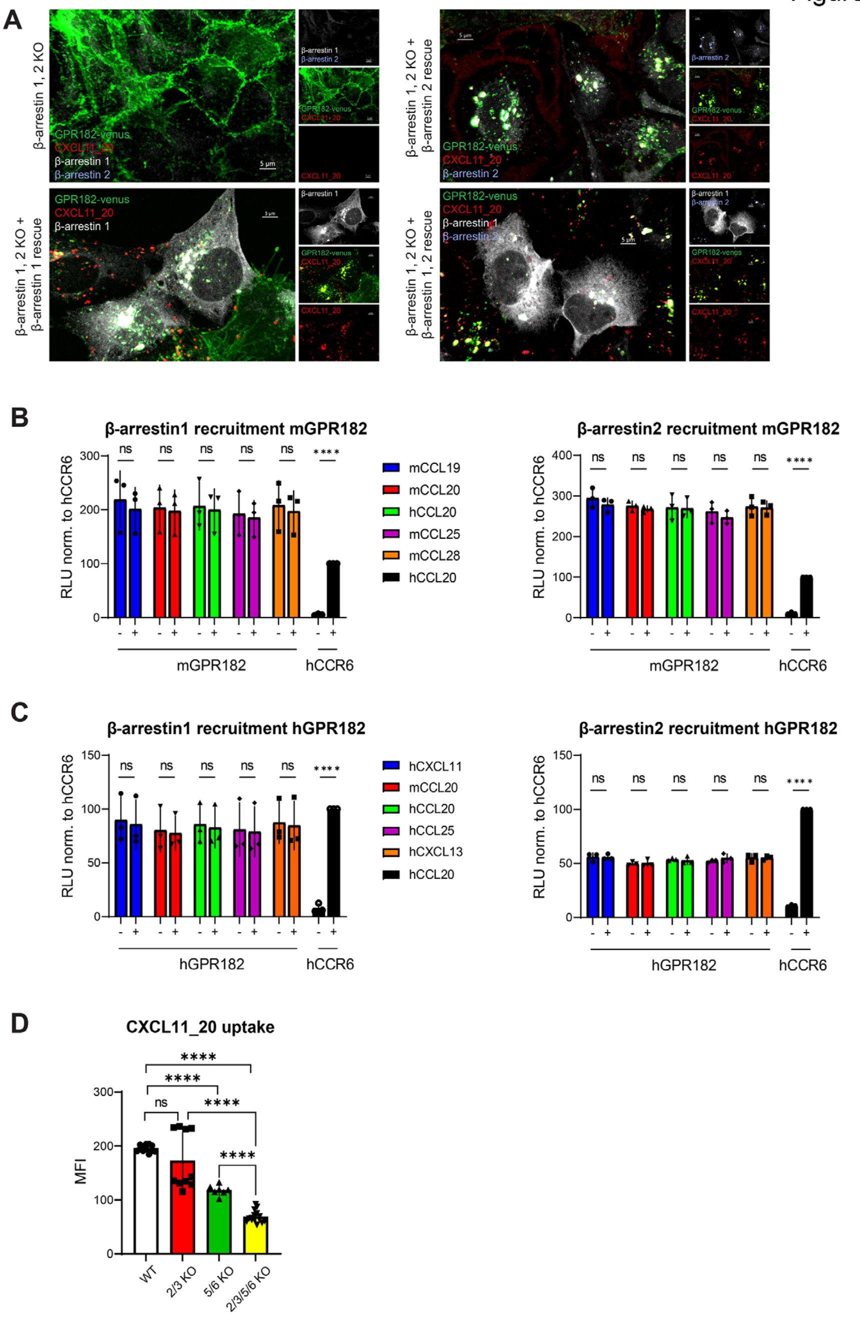

Fig S6 GPR182 requires β-arrestin recruitment for internalization and scavenging, but the C-terminus is dispensable. (**A**) Confocal images of CXCL11_20 (red) uptake and subcellular localization of GPR182-venus (green) in HEK293 β-arrestin 1,2 KO (left, upper panels). Expression of β-arrestin 1-ECFP (grey) rescues mGPR182 internalization and chemokine uptake (left, lower panels); β-arrestin 2-mCardinal (purple) expression rescues GPR182 internalization and chemokine uptake (right, upper panels); and (right lower panels) β-arrestin 1 (grey) and 2 (purple) concomitant rescue GPR812 (green) internalization and chemokine uptake (red). Data from one representative experiment out of three. Scale bars = 5μm. (**B-C**)) Comparison of steady-state interaction and chemokine-induced (300 nM)

recruitment of β-arrestin 1A-nLuc156 (left panels) and of β-arrestin2i1-nLuc156 (right panels) to (B) mGPR182-nLuc11 or (C) hGPR182-nLuc11 in HeLa cells. Means of measurements at three minutes before (-) and three minutes after (+) stimulation with 300 nM chemokine of three independent experiments are shown. Data were normalized to the maximal recruitment of β-arrestins to hCCR6-nLuc156. Error bars ± SEM. N = 3, unpaired two-tailed t- test; ns: not significant, ****$p < 0.0001$. (**D**) Quantification of mean fluorescence intensities (MFI) measured by confocal microscopy (determined with ImageJ) of internalized CXCL11_20-ATTO647 (100 nM) in HEK293 cells expressing mGPR182-venus (white bar), in the same cells with deletion of GRK 2/3 (red bar), deletion of GRK 5/6 (green bar), and deletion of GRK 2/3/5/6 (yellow bar). Data from one representative experiment, 4 fields of view were analyzed with at least 2 cells per field of view analyzed. Error bars ± SD. Unpaired two-tailed t-test, ns: not significant ****$p<0.0001$.

Figure S7

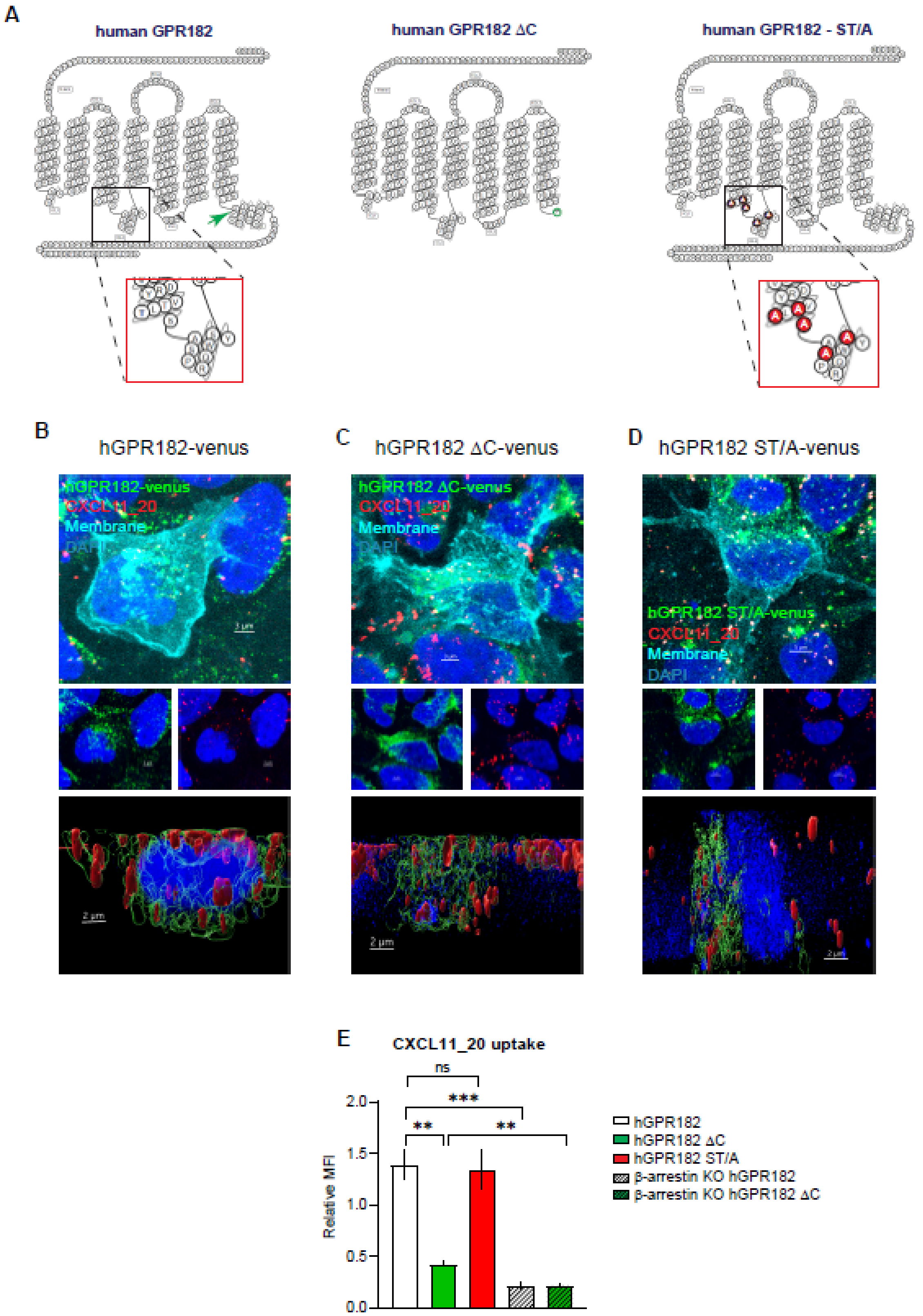
A
human GPR182
human GPR182 ΔC
human GPR182 - ST/A
B
hGPR182-venus
C
hGPR182 ΔC-venus
D
hGPR182 ST/A-venus
hGPR182-venus
CXCL11_20
Membrane
DAPI
hGPR182 ΔC-venus
hGPR182 ST/A-venus
2 µm
E
CXCL11_20 uptake
ns
***
**
**
Relative MFI
2.0
1.5
1.0
0.5
0.0
hGPR182
hGPR182 ΔC
hGPR182 ST/A
β-arrestin KO hGPR182
β-arrestin KO hGPR182 ΔC

Fig S7. hGPR182 does not require its C-terminus or serine/threonine residues in the second intracellular loop for receptor internalization and chemokine scavenging. (**A**) Schematic representation of amino acid structure of human GPR182 (left) with modifications annotated (enlarged red box for second intracellular loop (ICL2), green arrow for C-terminus deletion site); hGPR182 ΔC (middle); hGPR182 ST/A (right), with enlargement of S and T to A (red circles) substitutions in red box. (**B-D**) Confocal images showing expression of hGPR182-venus (B, green), or hGPR182ΔC-venus (C, green), or hGPR182 ST/A-venus (D, green), with CXCL11_20 uptake (red), plasma membrane labeling (Lck-mCherry, cyan) and DAPI nuclear staining (blue) in HEK293 cells. Middle panels show DAPI with mGPR182-venus (blue/green, left), or DAPI with CXCL11_20 (blue/red, right). Bottom panels depict 3D reconstruction showing chemokine internalization in endosomal like structures. Scale bars = 3μm and 5μm (top and middle panels), 2μm (bottom panels). (**E**) MFI of CXCL11_20 uptake relative to the fluorescence of Venus measured by FACS in $HEK293^{WT}$ cells expressing hGPR182-Venus (white bar); or hGPR182ΔC-Venus (green bar); or hGPR182 ST/A-Venus (red bar); or β-arrestin $1,2^{KO}$ cells expressing hGPR182-Venus (β-Arrestin KO hGPR182 hatched white bar); or hGPR182ΔC-Venus (β-Arrestin KO hGPR182ΔC, hatched green bar). MFI of surface bound CXCL11_20 was measured by incubating cells with chemokine at 17ºC and was subtracted from uptake at 37ºC. N = 3. Error bars ± SD. Unpaired two-tailed t-test, ns = not significant, ** $p<0.01$ ***$p<0.001$.

Figure S8

A

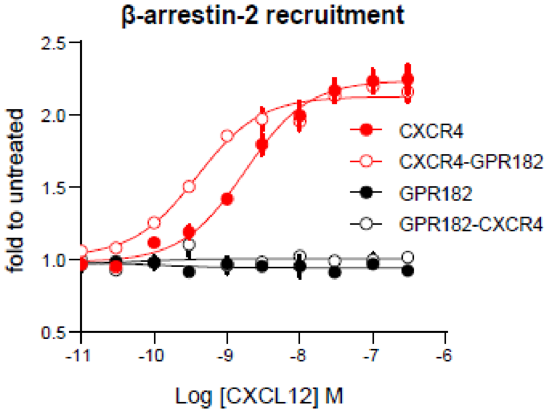

	CXCR4	CXCR4-GPR182	GPR182	GPR182-CXCR4
EC50	1.762e-009	3.850e-010	1.265e-010	5.313e-011

Fig S8. GPR182 interacts with β-arrestin in an unconventional way. (**A**) CXCL12-induced β-arrestin 2 recruitment to GPR182 (black, closed circles), CXCR4 (red, closed circles) and two chimeras corresponding to GPR182 (1-320) with the C-terminal tail of CXCR4 (303-352) (GPR182-CXCR4, black, open circles) or CXCR4 (1-302) with the C-terminal tail of GPR182 (321-404) (CXCR4-GPR182, red, open circles) monitored by NanoLuc complementation assay (NanoBiT). Data are expressed as fold change to chemokine untreated samples. N = 3 means ± SEM.

Figure S9

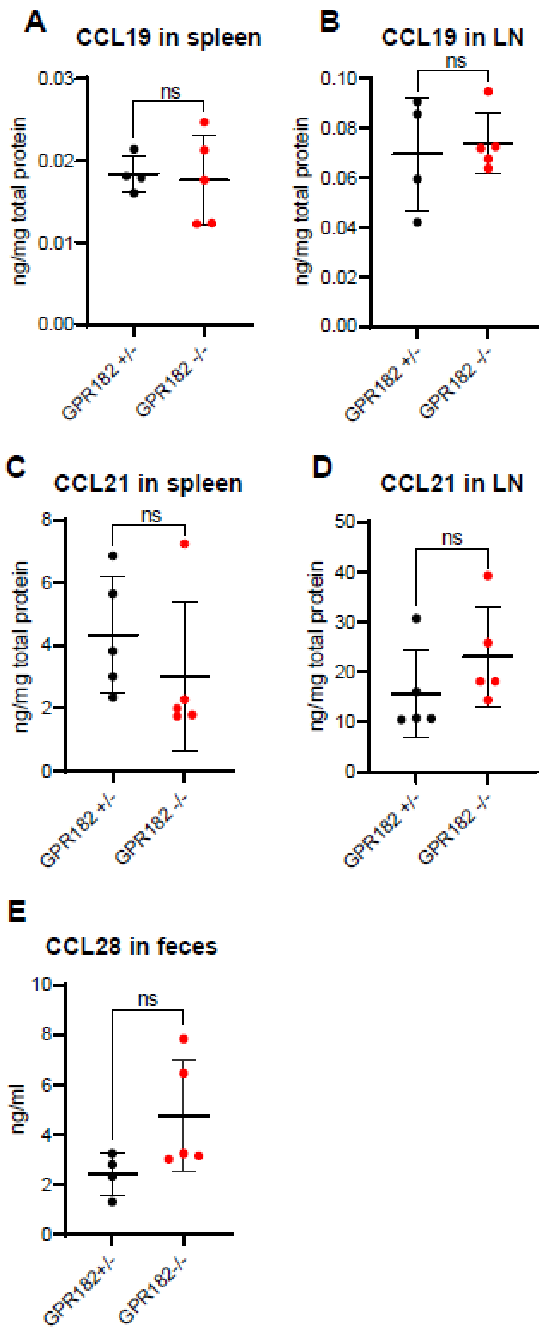

Fig S9. GPR182 does not regulate CCL19 and CCL21 levels in SLO and CCL28 in intestinal lumen. Interstitial CCL19 levels (**A**) in spleen and (**B**) in LN, CCL21 levels (**C**) in spleen and (**D**) in LN of $GPR182^{+/-}$ (black circles) and $GPR182^{-/-}$ (red circles) mice. Chemokine levels were normalized to total protein content. (**E**) CCL28 levels were measured in feces of $GPR182^{+/-}$ (black circles) and $GPR182^{-/-}$ (red circles) mice. (A-E) N = 4-5 per group. Error bars means ± SD (mean thick horizontal line). Unpaired two-tailed t-tests, ns = not significant.

Figure S10

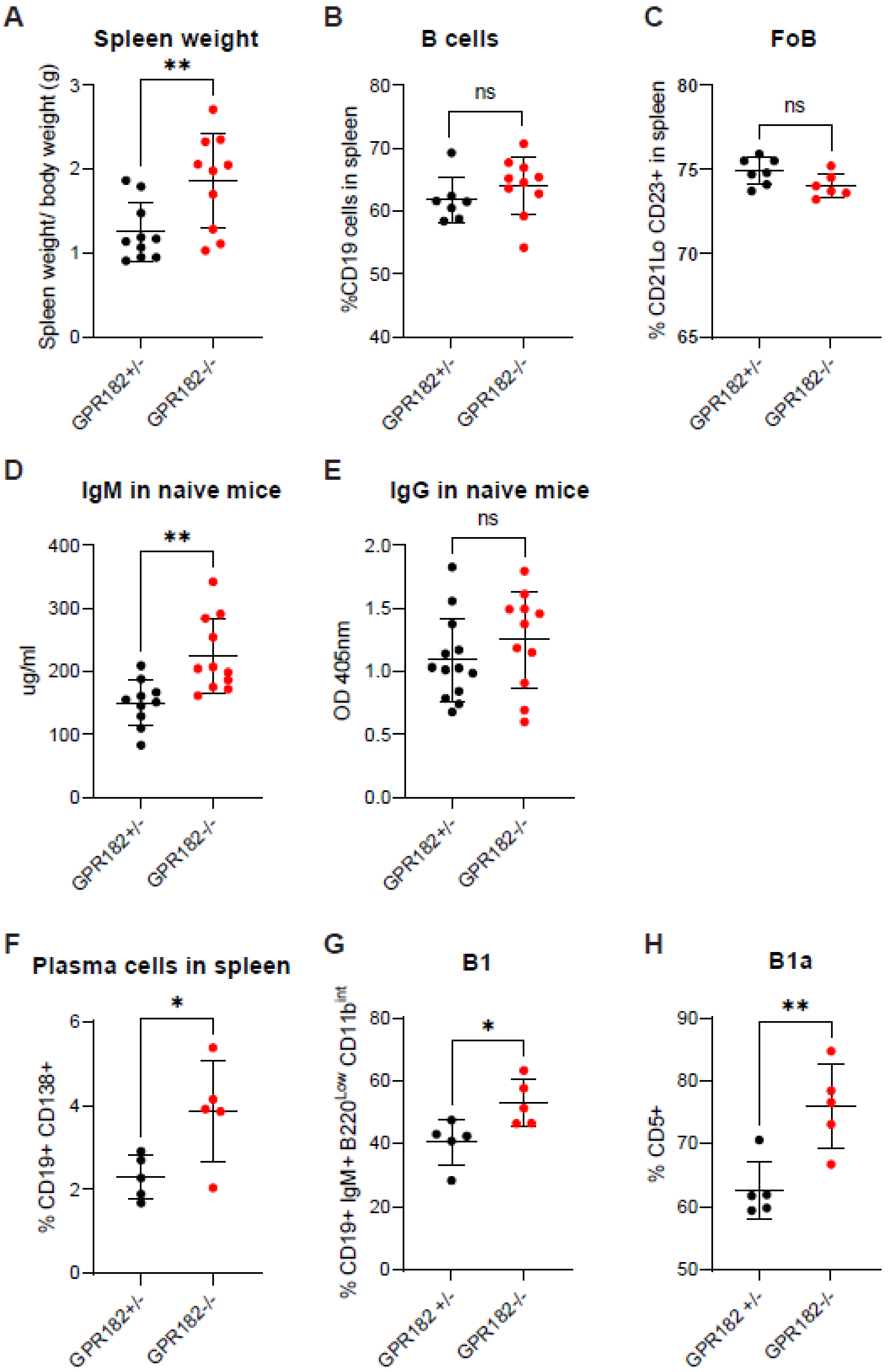
A
Spleen weight
Spleen weight/ body weight (g)
**
GPR182+/-
GPR182-/-
B
B cells
%CD19 cells in spleen
ns
C
FoB
% CD21Lo CD23+ in spleen
ns
D
IgM in naive mice
ug/ml
**
E
IgG in naive mice
OD 405nm
ns
F
Plasma cells in spleen
% CD19+ CD138+
*
G
B1
% CD19+ IgM+ B220Low CD11bint
*
GPR182 +/-
H
B1a
% CD5+
**

Fig S10. Immune phenotyping of GPR182$^{-/-}$ mice. (**A**) Spleen weight normalized to body weight. (**B**) % CD19^{+} cells of total splenocytes; (**C**) % CD21^{Low} CD23^{+} cells (FoB) of spleen B cells (CD19^{+} B220^{+}); IgM (**D**) and IgG (**E**) levels in serum of naïve GPR182$^{-/+}$ (black circles) and GPR182$^{-/-}$ (red circles) mice. (**F**) % CD138^{+} plasma cells of spleen B cells (CD19^{+} B220^{+}) of naïve GPR182$^{-/+}$ (black circles) and GPR182$^{-/-}$ (red circles) mice. (**G**) % Peritoneal B1 (CD19^{+} IgM^{+} B220^{Low} CD11b^{int}) and (**H**) B1a cells (CD5^{+}) of naïve GPR182$^{-/+}$ (black circles) and GPR182$^{-/-}$ (red circles) mice. N=5-11/group Error bars means ± SD, means thick horizontal line. Unpaired two-tailed t-test *p<0.05, **p<0.01), ns: not significant.

Figure S11

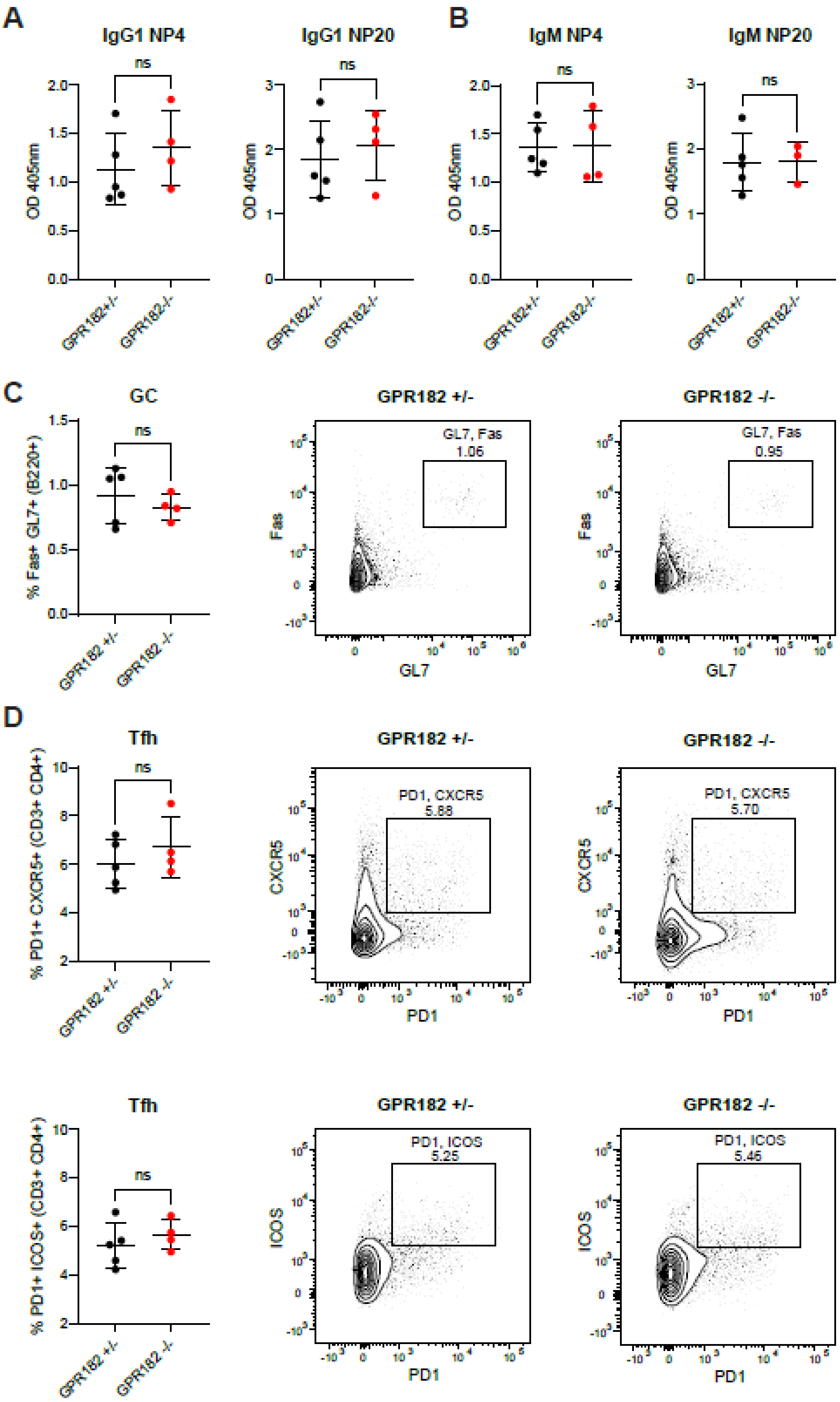

A
IgG1 NP4
IgG1 NP20
B
IgM NP4
IgM NP20
ns
OD 405nm
GPR182+/-
GPR182-/-
C
GC
% Fas+ GL7+ (B220+)
GPR182 +/-
GPR182 -/-
GL7, Fas
1.06
0.95
Fas
GL7
D
Tfh
% PD1+ CXCR5+ (CD3+ CD4+)
PD1, CXCR5
5.88
5.70
CXCR5
PD1
% PD1+ ICOS+ (CD3+ CD4+)
PD1, ICOS
5.25
5.46
ICOS

Fig S11. GC and antibody responses to T-dependent antigens are normal in GPR182$^{-/-}$ mice. Day 14 antigen-specific IgG1 (**A**) and IgM (**B**) levels in NP-KLH immunized mice, high affinity (NP4, left) and low affinity (NP20, right). (**C**) Germinal center B cell percentage following immunization (Fas^{+} GL7^{+}), with gating strategy (right panels). (**D**) T follicular helper cell percentages following immunization stained for different markers PD1^{+} CXCR5^{+}, (upper panels) PD1^{+} ICOS^{+} (lower panels), with gating strategy (right panels). N=4-5 per group. Error bars means ± SD (mean thick horizontal line). Unpaired two-tailed t-tests, ns = not significant.

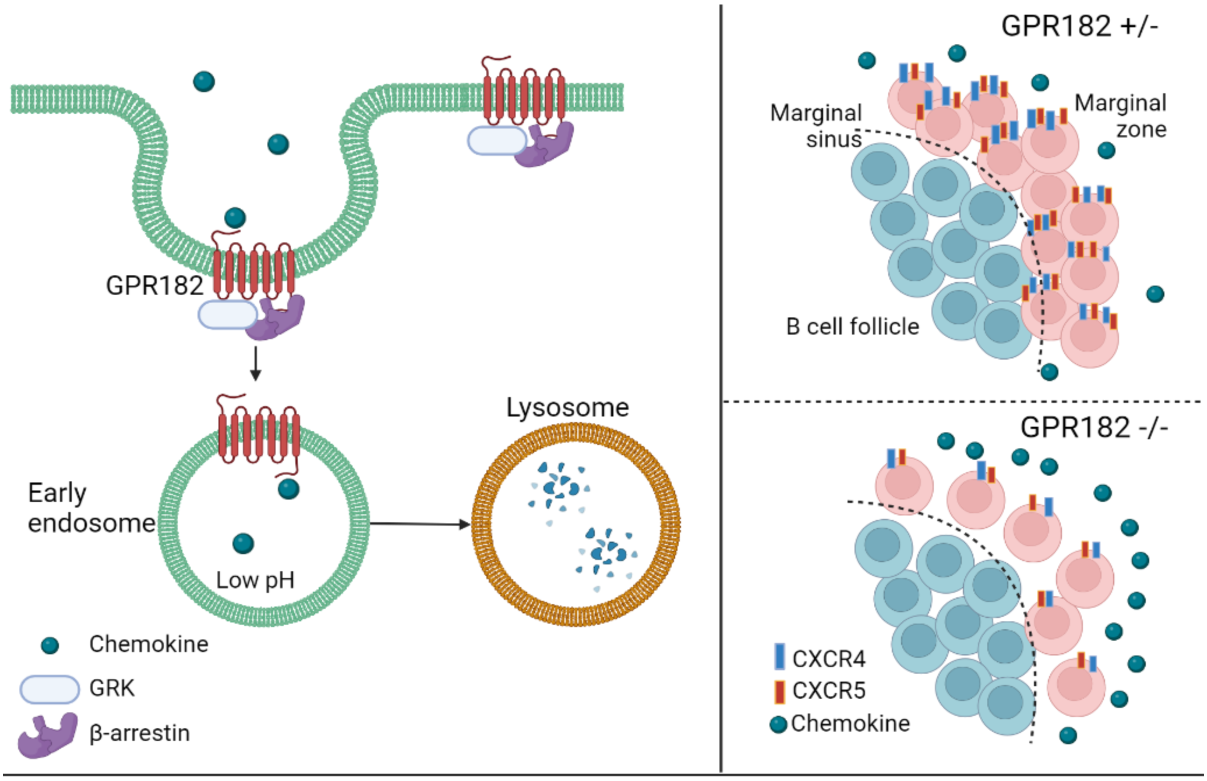

Fig S12. Schematic summary of the observations. GPR182 at the plasma membrane is precoupled to β-arrestin and prone to spontaneous internalization which depends on GRK activity. Internalized GPR182 localizes to endosomes and delivers the chemokines for lysosomal degradation sequestering chemokines from the outer cellular environment. GPR182 maintains moderate levels of CXCL12 and CXCL13 in the interstitium of the spleen. When the receptor is genetically ablated the levels of these chemokines rise leading to the down regulation of the complementary receptors CXCR4 and CXCR5 respectively on splenic B cells which may cause a decrease in marginal zone B cells leading to a reduced T cell independent immune response.
